# Improved production of dibenzocyclooctadiene lignans in the elicited microshoot cultures of *Schisandra chinensis* (Chinese magnolia vine)

**DOI:** 10.1007/s00253-017-8640-7

**Published:** 2017-11-27

**Authors:** Agnieszka Szopa, Adam Kokotkiewicz, Agata Król, Maria Luczkiewicz, Halina Ekiert

**Affiliations:** 1 Department of Pharmaceutical Botany, Jagiellonian University, Collegium Medicum, ul. Medyczna 9, 30-688 Kraków, Poland; 2 Department of Pharmacognosy, Faculty of Pharmacy, Medical University of Gdańsk, al. gen. J. Hallera 107, 80-416 Gdańsk, Poland

**Keywords:** Elicitation, Temporary-immersion bioreactor, *Schisandra* lignan production, Biotic elicitors, Abiotic elicitors, In vitro cultures

## Abstract

Dibenzocyclooctadiene lignans are a specific group of secondary metabolites that occur solely in *Schisandra chinensis*. The aim of the presented work was to boost the accumulation of lignans in the agitated microshoot cultures of *S. chinensis*, using different elicitation schemes. The experiments included testing of various concentrations and supplementation times of cadmium chloride (CdCl_2_), chitosan (Ch), yeast extract (YeE), methyl jasmonate (MeJa), and permeabilizing agent—dimethylsulfoxide (DMSO). After 30 days, the microshoots were harvested and evaluated for growth parameters and lignan content by LC-DAD method. The analyses showed enhanced production of lignans in the elicited *S. chinensis* microshoots, whereas the respective media samples contained only trace amounts of the examined compounds (< 5 mg/l). Elicitation with CdCl_2_ caused up to 2-fold increase in the total lignan content (max. ca. 730 mg/100 g DW after the addition of 1000 μM CdCl_2_ on the tenth day). Experiments with chitosan resulted in up to 1.35-fold increase in lignan concentration (max. ca. 500 mg/100 g DW) after the supplementation with 50 mg/l on the first day and 200 mg/l on the tenth day. High improvement of lignan production was also recorded after YeE elicitation. After the elicitation with 5000 mg/l of YeE on the first day of the growth period, and with 1000 and 3000 mg/l on the 20th day, the lignan production increased to the same degree—about 1.8-fold. The supplementation with 1000 mg/l YeE on the 20th day of the growth cycle was chosen as the optimal elicitation scheme, for the microshoot cultures maintained in Plantform temporary immersion system—the total content of the estimated lignans was equal to 831.6 mg/100 g DW.

**Electronic supplementary material:**

The online version of this article (10.1007/s00253-017-8640-7) contains supplementary material, which is available to authorized users.

## Introduction

*Schisandra chinensis* (Turcz.) Baill., Chinese magnolia vine (*Schisandraceae*), is a climbing plant, naturally occurring in the countries of Eastern Asia, whereas in European countries, it grows mainly as an ornamental shrub (Panossian and Wikman 2008; Szopa et al. 2016a; Szopa et al. 2017a). The raw material of *Schisandra* fruits, *Schisandrae chinensis fructus* (chin. běi wǔ wèi zi; literally “five-flavor berry”), has been used for therapeutic purposes in traditional Chinese medicine and has successfully been included in pharmacopoeial monographs of Asian and European countries as well as in the USA Pharmacopoeia and the International Pharmacopoeia, printed by WHO (World Health Organization 2007; European Directorate for the Quality of Medicines. 2017; Szopa et al. 2017a). Numerous therapeutic properties of *Schisandra* fruit extracts, as well as its individual compounds, have been confirmed by the scientific research, carried out with the use of in vivo and in vitro models. The studies indicate that *S. chinensis* has a positive effect on liver functioning and stimulates cardiovascular, respiratory, and central nervous systems. The therapeutic properties of Chinese magnolia vine include anticancer, immunostimulant, and adaptogenic (Mocan et al. 2016; Szopa et al. 2016a; Szopa et al. 2017a). These activities are related to the presence of the dibenzocyclooctadiene lignans, known as “*Schisandra* lignans” (Figure S1), due to the fact that their occurrence is limited to *S. chinensis* (Fuss 2004; Opletal et al. 2004; Lu and Chen 2009).

The dominant lignans that can be distinguished out of over 40 present in *S. chinensis* are as follows: schisandrin; gomisins A, C, F and N; deoxyschisandrin; and *γ*-schisandrin. The extracts of *S. chinensis* fruit have been used for the manufacture of medical products, food supplements, or cosmetics (Szopa et al. 2016a). Given the fact that the lignans of *S. chinensis* are therapeutically unique compounds, the research on alternative methods of their natural resource-independent acquisition has been performed. The methods of the chemical synthesis of *Schisandra* lignans have been developed; however, these had limited success due to their complex stereochemistry (Shi et al. 2009). The other alternative is plant cell culture which has so far been employed for the production of several biologically active secondary metabolites, including lignans. Moreover, it has been reported that in vitro techniques enable to enhance the production of lignans by means of elicitation, addition of biosynthetic precursors, and immobilization (Angelova et al. 2006; Verpoorte et al. 2002; Capote 2012; Ramirez-Estrada et al. 2016). Nevertheless, it should be noted that the so far conducted studies focused on the production of aryltetralin lignans in the in vitro cultures of various species of *Podophyllum* sp. and *Linum* sp. (Petersen and Alfermann 2001; Arroo et al. 2002; Koulman et al. 2003; Malik et al. 2014). On the other hand, there are only few studies dealing with the accumulation of *Schisandra* lignans under cell culture conditions (Fuss 2004). In our previous studies, agar, stationary liquid, and agitated microshoot cultures of *S. chinensis* were demonstrated to produce substantial amounts of dibenzocyclooctadiene lignans (Szopa et al. 2016b). Moreover, in our latest work, we optimized the production process of these compounds in different types of laboratory scale bioreactors (Szopa et al. 2017b).

The current work was aimed at determining the effect of elicitation on the accumulation of dibenzocyclooctadiene lignans in *S. chinensis* microshoots. The agitated cultures were supplemented with abiotic elicitor: cadmium chloride (CdCl_2_), and biotic elicitors: chitosan (Ch), yeast extract (YeE), methyl jasmonate (MeJa), and the permeabilizing agent—dimethylsulfoxide (DMSO). The elicitor-treated cultures were evaluated for growth (fresh weight [FW], dry weight [DW], growth index [Gi]) and lignan accumulation. The experiments included different concentrations and application times of elicitors. The most effective elicitation scheme was subsequently applied to bioreactor-grown microshoots.

## Materials and methods

### Basic agar culture

The microshoot cultures of *Schisandra chinensis* (Turcz.) Baill. (Szopa et al. 2016b), grown on agar Murashige and Skoog (MS) medium (Murashige and Skoog 1962) and supplemented with 30 g/l sucrose, 3.0 mg/l BA, and 1.0 mg/l NAA (further referred to as MS_Sch_ medium), were used for the experiments. The cultures are deposited in the Department of Pharmaceutical Botany, Jagiellonian University, Poland. The cultures were maintained at 25 ± 2 °C under constant light (white fluorescent tubes, 36 W, 88 ± 8 μmol m^−2^ s^−1^, Philips, Amsterdam, Netherlands) and subcultured at 60-day intervals.

### Agitated cultures

For the agitated culture initiation, 1.5 g *S. chinensis* microshoots (see the “Basic agar culture” section), grown on MS_Sch_ medium for 60 days, were placed into 125 ml Erlenmeyer flasks, filled with 50 ml liquid MS_Sch_ medium, and closed with silicone sponge stoppers (Carl-Roth, Karlsruhe, Germany). The cultures were maintained on the rotary shaker at 120 rpm (INNOVA 2300, Eppendorf, Enfield, US-CT), under light and temperature conditions described beforehand (see the “Basic agar culture” section).

### Bioreactor cultures

*S. chinensis* shoots were grown for 30 days in Plantform temporary immersion system (Plant Form AB, Lomma, Sweden), as previously described (Szopa et al. 2017b). The bioreactor was inoculated at 15/500 microshoots to medium ratio (g/ml). The immersion cycle was set to 5 min every 1.5 h, at 1.0 vvm aeration rate.

### In vitro culture reagents

The culture media were prepared using Sigma-Aldrich (St. Louis, MO, USA) reagents and type I water (Elix/Synergy system, Merck-Millipore, Billerica, MA, USA). Stock aqueous solutions of cadmium chloride (CdCl_2_) (100, 10, 1, and 0.1 mM, reagent grade, POCH, Gliwice, Poland) and yeast extract (YeE) (250, 150, 50, and 5 g/l, plant cell culture tested, Sigma-Aldrich) for elicitor treatments were steam sterilized (120 °C, 20 min, 1 bar) prior to use. The stock solution of deacetylated crab shell chitosan (Ch) (5.0 g/l, Sigma-Aldrich) was prepared by dissolving 0.5 g of chitosan in 20 ml of hot aqueous solution (5% *w*/*w*) of hydrochloric acid (HCl; Merck, Darmstadt, Germany). The obtained solution was diluted to 50 ml with water, its pH was adjusted to 5.8, using 1 M sodium hydroxide, and diluted with water to the final volume of 100 ml. Experimental stock solutions of chitosan (2.5, 1.25, and 0.625 g/l) were prepared by diluting the 5.0 g/l solution with neutralized hydrochloric acid solution (20 ml of 5% *v*/*w* aqueous hydrochloric acid diluted with water to 50 ml, adjusted to pH 5.8, using 1 M sodium hydroxide, and diluted to the final volume of 100 ml). The stock solutions of chitosan as well as neutralized HCl solution (used for the control experiments in the elicitor treatments) were steam sterilized (120 °C, 20 min, 1 bar) prior to use.

### Elicitor treatments

#### Elicitation of the agitated microshoot cultures

Sterile stock solutions of elicitors (prepared as described in the “In vitro culture reagents” section) were added to the agitated microshoot cultures (established as described in the “Agitated cultures” section) on the first, 10th, and 20th day of the 30-day growth period. The stock solutions of cadmium chloride (CdCl_2_), yeast extract (YeE), and chitosan (Ch) were added at 0.5, 1.0, and 2.0 ml per flask, respectively, yielding the final concentrations of 1000, 100, 10, and 1 μM (CdCl_2_); 5000, 3000, 1000, and 100 g/l (YeE); and 200, 100, 50, and 25 mg/l (Ch) in the growth medium. The control groups constituted the agitated microshoots without elicitor treatment (all experiments), as well as the cultures supplemented with neutralized HCl solution (see the “In vitro culture reagents” section; chitosan experiments only). After 30 days, the microshoots and media samples were collected, freeze-dried (LYOVAC GT2 apparatus, Finn-Aqua Santasolo-Sohlberg, Tuusula, Finland), and subjected to phytochemical analysis.

### Elicitation of the bioreactor-grown microshoots cultures

For the Plantform bioreactor experiments, the elicitation protocol with 1000 mg/l of YeE, supplemented on the 20th day of the growth period, was applied. For elicitation, one of the side hose nipples of Plantform bioreactor was used as an inlet port. The microshoots and media samples were collected on the 30th day of the experiment. The samples were freeze-dried and subjected to phytochemical analysis.

### Calculating the growth parameters

The growth parameters were expressed as fresh and dry weight (FW and DW, determined before and after freeze-drying, respectively), as well as growth index (Gi), calculated according to the formula: Gi = [(FW1 − FW0)/FW1] × 100, where FW1 is the fresh weight of microshoots at the end of the experiment and FW0 is the fresh weight of the *inoculum.*

### Extraction, separation, and quantification of *Schisandra* lignans

The lyophilized (LYOVAC GT2 apparatus, Finn-Aqua Santasolo-Sohlberg, Tuusula, Finland) biomass samples, collected after the 30th day of the growth periods (0.5 g of DW (dry weight)) from each of four experimental series, were sonicated (Polsonic 3, Warszawa, Poland) at a frequency of 40 kHz and an intensity of 160 W, with methanol (2 × 50 ml) at 30 °C. The lyophilized media samples (40 ml) were extracted in 5 ml of methanol. For the estimation of lignan contents, quantification was carried out by liquid chromatography with diode array detection (LC-DAD), as described previously (Zhang et al. 2009; Szopa et al. 2016b; Szopa et al. 2017b).

### Statistical analysis

The experiments have been repeated thrice. The results were presented as mean ± standard deviation (SD). The STATISTICA version 12 PL software package (StatSoft) was used for the analysis. The results of total lignan contents were compared with the one-way analysis of variance (one-way ANOVA). For comparison and contrast between different groups, post hoc Tukey HSD (honestly significant difference) test was used.

## Results

### Preliminary experiments

In the course of preliminary experiments, the biomass was elicited with methyl jasmonate (MeJa) at 50, 100, and 200 μM and cadmium chloride (CdCl_2_) at 2.5, 5, 10, and 20 mM. Additionally, dimethylsulfoxide (DMSO) at 0.2, 2, 4, and 8% *v/v* was tested as a permeabilizing agent. All tested agents were added on the 23th or the 27th day, and the experiment was run for 30 days. The collected shoots were evaluated for growth and lignan content, and the results were included as supplementary Figures S2–S4 and Tables S1–S3 (Online resource 1). In all elicited biomass extracts, fourteen dibenzocyclooctadiene lignans were detected: schisandrin, gomisin A, gomisin G, schisantherin A, schisantherin B, schisanthenol, deoxyschisandrin, γ-schisandrin, schisandrin C, angeoyl/tigloylgomisin H, angeoyl/tigloylgomisin Q, schisandrin B, benzoylgomisin P, and schisantherin D (Tables S1–S3).

Medium supplementation with DMSO caused the decrease in intracellular lignan content. The greatest decrease of total lignan contents was observed for the addition of 40 ml/l of DMSO on 27th (239.9 mg/100 g DW) and 23rd (312.9 mg/100 g DW) days of culture growth. This relation was not accompanied by the increase of the lignan concentration in the growth medium (Table S1). The detected amounts in the media samples were low, below 5 mg/l. The DMSO also restrains the biomass growth (Figure S2).

The experiments with MeJa showed variable influence of this elicitor on *S. chinensis* microshoot growth, the Gi factor oscillated between 254 and 346% (Figure S3). Depending on concentration, MeJa either moderately decreased or did not affect the accumulation of lignans (max. total content 300–400 mg/100 g DW) (Table S2). The highest contents, 427.8 mg/100 g DW, were detected after the addition of 50 μM of MeJa on the 23rd day. As in the case of DMSO supplementation, the media samples showed trace presence of lignans.

Among the preliminary experiments, the best results were obtained by CdCl_2_ elicitation: its addition to the growth medium caused up to 2-fold increase in lignan content (597.3 mg/100 g DW and 652.5 mg/100 g DW, after the addition of 2.5 or 20 mM CdCl_2_ on the 23rd day, respectively) (Table S3). In the applied concentration range, CdCl_2_ was toxic for the shoots, as they showed visible signs of necrosis (darkening, medium browning). The growth rates, however, were not lower than in the control group (Figure S4). The supplementary experiment, involving the application of CdCl_2_ at 1.25–2500 μM on the first day of the growth cycle demonstrated that growth inhibition occurs at ≥ 125 μM CdCl_2_ (Figure S5). Low concentrations of cadmium ions (1.25 μM), on the other hand, did not negatively affect culture growth while exerting moderately stimulating effect on lignan accumulation (Table S4). The highest total lignan content was obtained after the addition of 2500 μM of MeJa. The respective media samples collected after elicitations with CdCl_2_ showed only trace amounts of the examined compounds (< 5 mg/l).

### The influence of elicitation on microshoots’ growth and morphology

In the applied concentration range (see the “Elicitor treatments” section), the elicitors did not change microshoots’ morphology. However, the shoots elicited with the highest doses of the elicitors were brownish and darkish, compared to the control group.

**Fig. 1:**
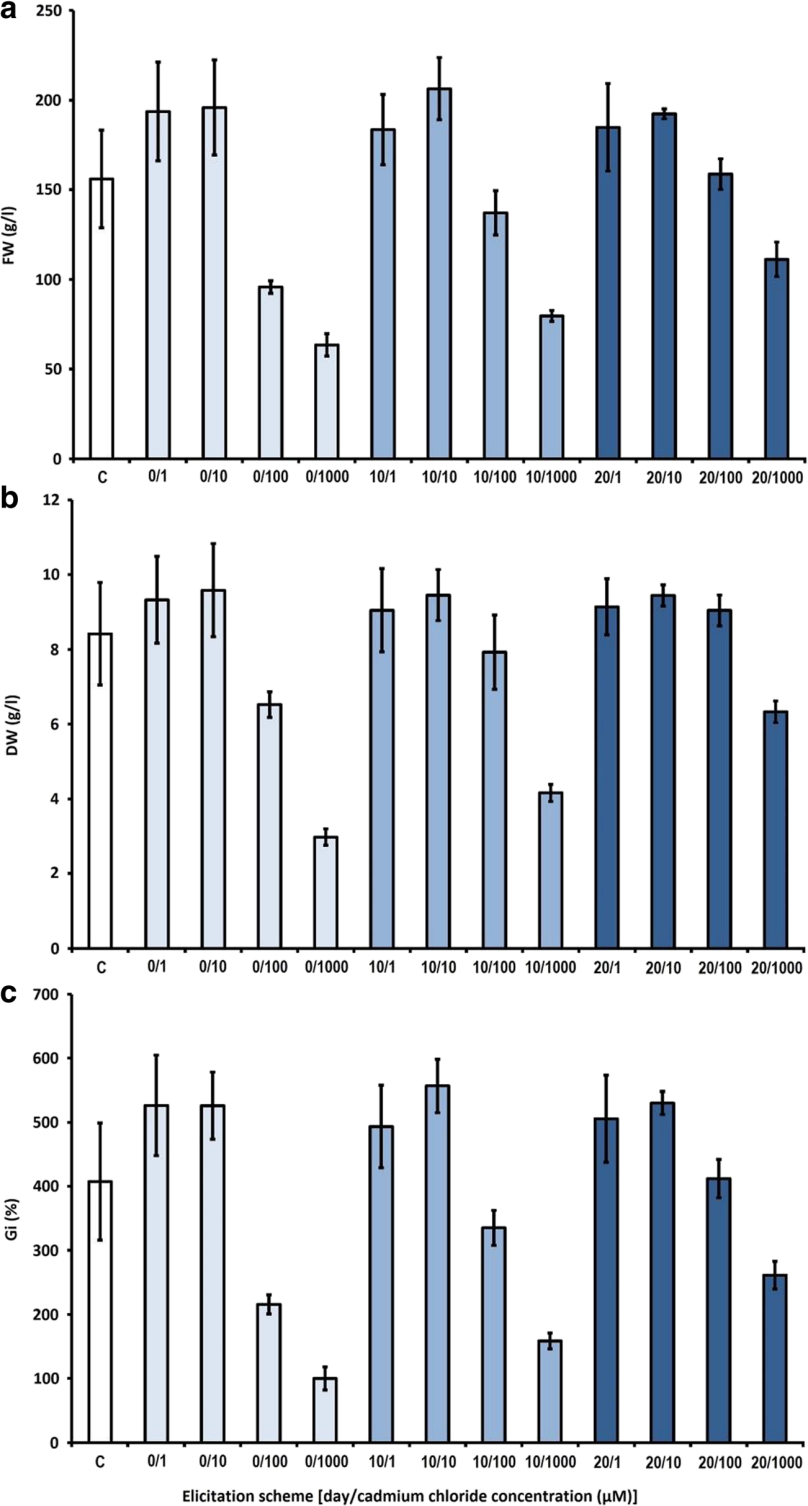
The effect of cadmium chloride on biomass growth in agitated shoot cultures of *S. chinensis*. **a** Fresh weight (FW). **b** Dry weight (DW). **c** Growth index (Gi). Symbols used: C—control group without cadmium chloride treatment. The cultures were grown for 30 days

Elicitation with the lower concentrations, 1 and 10 μM of CdCl_2_, had a positive effect on the biomass growth, irrespective of the time of elicitation. The calculated Gi factors ranged from 493.4 to 556.7%, in comparison with 407.4% recorded for the control samples. The higher concentrations, 100 and 1000 μM of CdCl_2_ supplemented on the first day of the growth period, noticeably decreased the growth increments. The Gi factor for the biomass elicited with 1000 μM CdCl_2_ on the 20th day was about four times lower than in the control samples (Fig. 1).

**Fig. 2:**
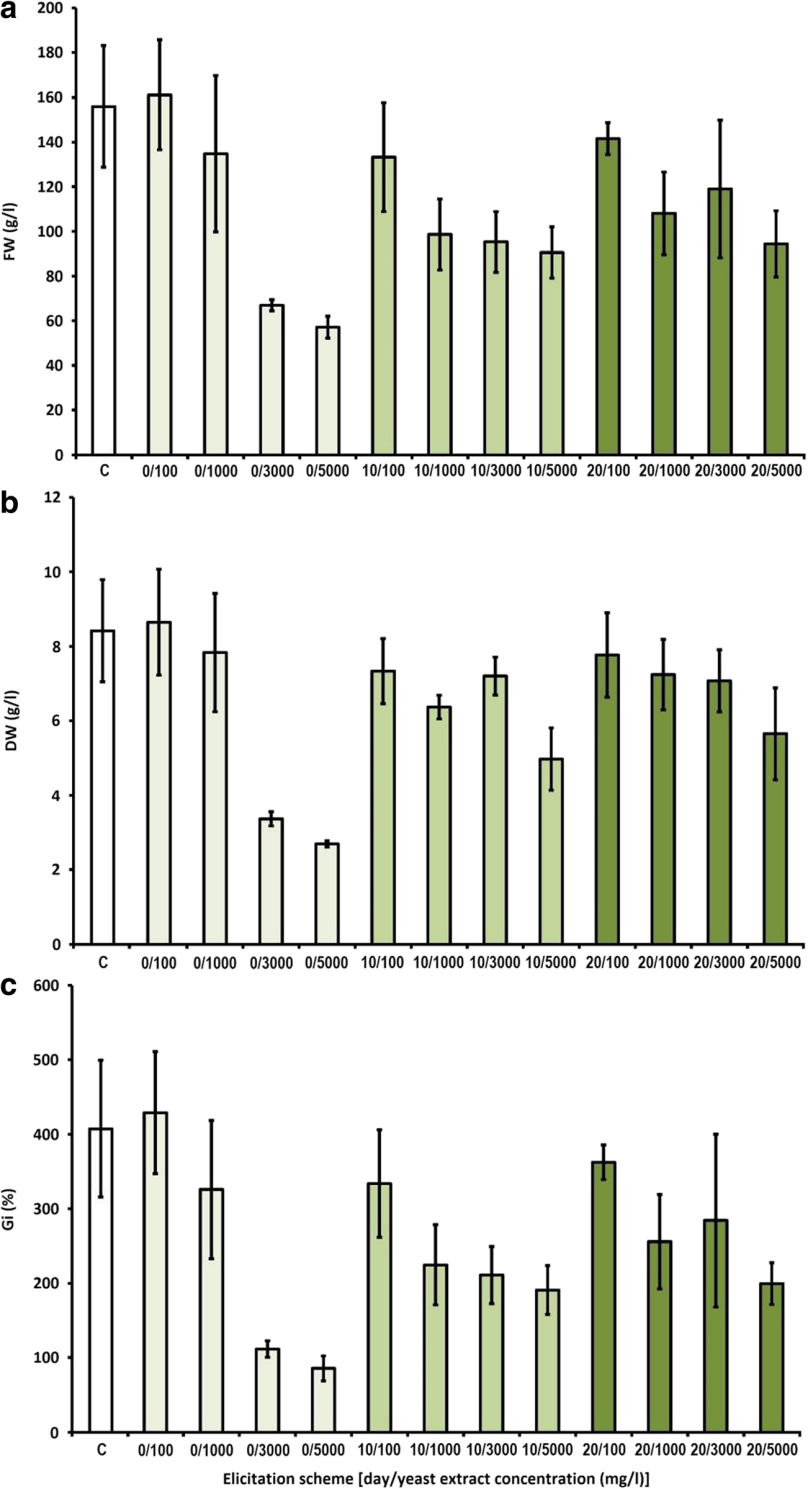
The effect of yeast extract on biomass growth in agitated shoot cultures of *S. chinensis*. **a** Fresh weight (FW). **b** Dry weight (DW). **c** Growth index (Gi). Symbols used: C—control group without yeast extract treatment. The cultures were grown for 30 days

The YeE added at 100 mg/l slightly influenced the biomass growth, with the Gi index (333.7–428.9%) comparable to the control samples (407.4%). All other YeE concentrations tested remarkably decreased the growth rates. The most noticeable decrease in Gi factor was observed after the addition of 3000 and 5000 mg/l YeE on the first day of the growth period (Fig. 2).

**Fig. 3:**
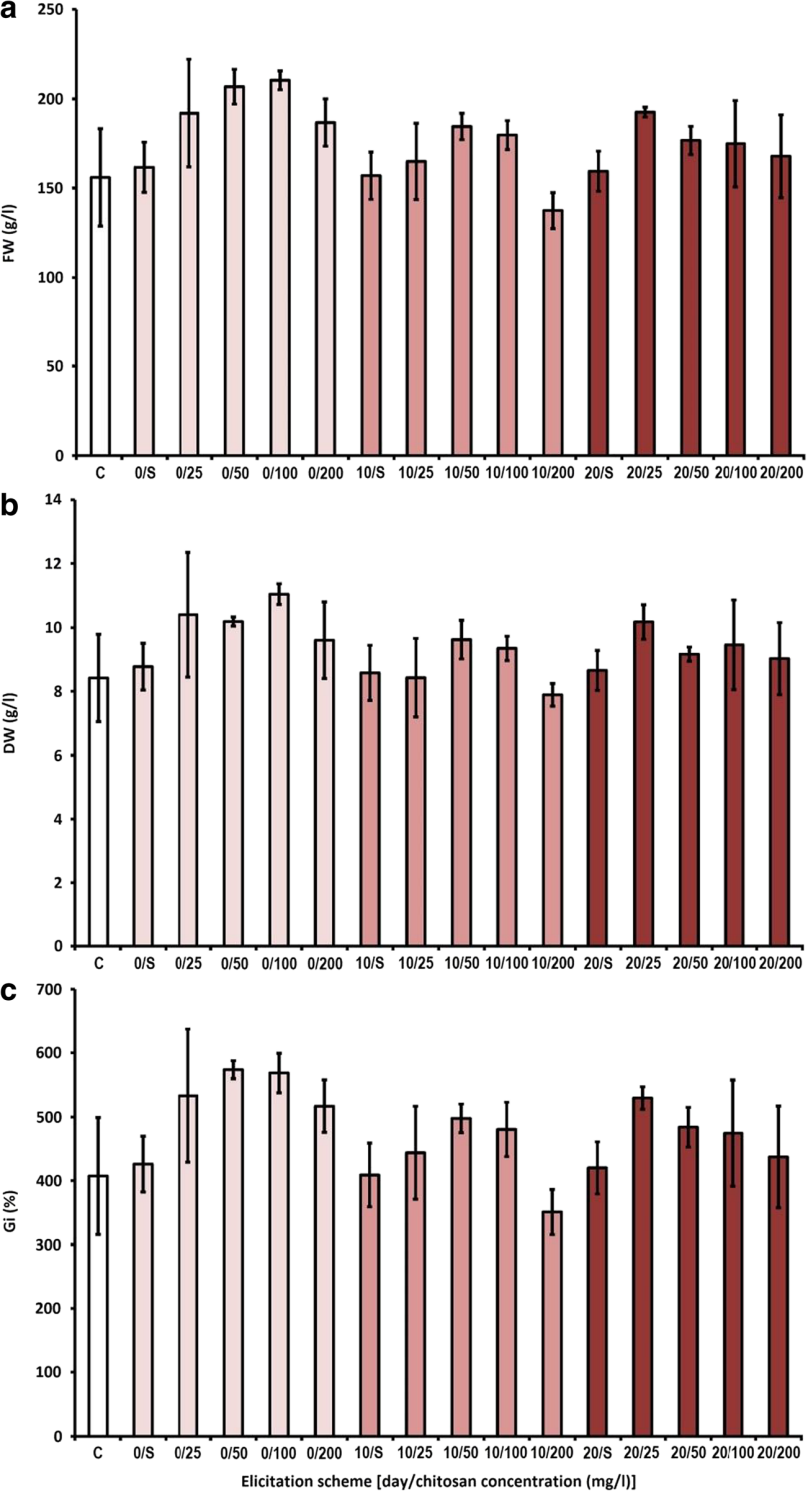
The effect of chitosan on biomass growth in agitated shoot cultures of *S. chinensis*. **a** Fresh weight (FW). **b** Dry weight (DW). **c** Growth index (Gi). Symbols used: C—control group without chitosan treatment, S—control group without chitosan treatment, supplemented with neutralized hydrochloric acid solution. The cultures were grown for 30 days

The elicitation with chitosan, in the applied concentration range (25–200 mg/l), did not negatively affect the microshoot growth. Regardless of the supplementation time, the growth indices of the chitosan-supplemented shoots were similar (or higher) to those of the control samples. The highest Gi value was noted for supplementation with 50 and 100 mg/l Ch on the first day of the experiment (573.9 and 568.7% respectively, Fig. 3).

### The effect of elicitation on the accumulation of lignans in the agitated microshoot cultures

The amounts of the main *Schisandra* lignans were estimated by chromatographic method in the methanolic extracts from the elicited microshoots, collected after 30-day growth periods. During the individual experiments, four dilutions of each elicitor were added, successively, on the first, 10th, and 20th days of the growth period. The elicitors and their concentrations were selected basing on the preliminary experiments, described in the supplementary data and above. In all samples irrespectively of the applied elicitation strategy, 14 dibenzocyclooctadiene lignans were estimated: schisandrin, gomisin A, gomisin G, schisantherin A, schisantherin B, schisanthenol, deoxyschisandrin, *γ*-schisandrin, schisandrin C, angeoyl/tigloylgomisin H, angeoyl/tigloylgomisin Q, schisandrin B, benzoylgomisin P, and schisantherin D (Tables 1, 2, 3, and 4). In the all analyzed samples, the main compounds were schisandrin, gomisin A, deoxyschisandrin, angeloyl/tigloylgomisins Q and H, and benzoylgomisin P. In the all experimental media samples, only the traces of the studied lignans were detected.

**Table 1:** Production [mg/100 g DW ± SD] of dibenzocyclooctadiene lignans in agitated microshoot cultures of *S. chinensis* elicited with cadmium chloride - CdCl_2_ (*n* = 3)

Lignans	Control	Elicitation scheme [day added/cadmium chloride ± CdCl_2_ concentration (μM)]											
		0/1	0/10	0/100	0/1000	10/1	10/10	10/100	10/1000	20/1	20/10	20/100	20/1000
Schisandrin	80.5 ± 8.0	103.5 ± 3.5	71.7 ± 8.2	67.0 ± 3.4	131.5 ± 7.3	84.4 ± 9.1	77.7 ± 5.3	93.3 ± 3.4	183.6 ± 9.1	87.0 ± 3.7	78.4 ± 7.3	70.2 ± 1.2	139.1 ± 5.3
Gomisin A	52.8 ± 7.5	73.8 ± 6.2	43.1 ± 5.9	39.6 ± 4.8	70.6 ± 3.6	50.2 ± 1.6	50.5 ± 1.9	65.9 ± 3.2	115.9 ± 17.0	64.2 ± 3.1	55.6 ± 6.8	52.5 ± 1.0	94.2 ± 7.4
Gomisin G	3.9 ± 0.7	2.7 ± 0.1	2.6 ± 0.1	2.3 ± 0.4	5.1 ± 0.4	1.6 ± 0.9	1.8 ± 0.7	3.0 ± 0.1	9.3 ± 0.5	2.0 ± 0.2	1.9 ± 0.1	1.6 ± 0.2	4.1 ± 1.0
Schisantherin A	1.8 ± 0.2	1.9 ± 0.1	1.1 ± 0.1	1.2 ± 0.1	3.9 ± 0.4	1.1 ± 0.1	1.4 ± 0.1	1.6 ± 0.0	5.5 ± 0.1	1.6 ± 0.0	1.4 ± 0.1	1.2 ± 0.1	3.1 ± 0.6
Schisantherin B	10.8 ± 0.2	7.9 ± 0.3	5.2 ± 0.4	8.8 ± 0.2	9.6 ± 0.6	5.5 ± 0.2	6.0 ± 0.2	6.6 ± 0.2	13.0 ± 1.1	6.8 ± 0.1	5.9 ± 0.2	6.7 ± 0.2	8.8 ± 0.5
Schisanthenol	2.5 ± 0.2	2.4 ± 0.1	2.1 ± 0.3	2.3 ± 0.1	5.7 ± 0.4	2.4 ± 0.2	2.4 ± 0.1	2.7 ± 0.1	11.1 ± 0.7	2.6 ± 0.4	2.4 ± 0.1	2.1 ± 0.3	7.0 ± 0.4
Deoxyschisandrin	34.6 ± 3.1	55.8 ± 1.8	36.4 ± 4.7	39.4 ± 1.6	57.7 ± 7.0	34.3 ± 1.1	40.0 ± 1.2	44.5 ± 0.9	63.1 ± 5.4	45.3 ± 1.4	40.1 ± 1.3	32.6 ± 2.5	61.4 ± 3.8
γ-Schisandrin	4.7 ± 0.6	7.0 ± 0.2	4.5 ± 0.3	5.2 ± 0.3	6.5 ± 0.8	4.6 ± 0.4	4.9 ± 0.4	5.8 ± 0.3	7.4 ± 0.3	5.8 ± 0.3	5.0 ± 0.5	4.4 ± 0.2	7.5 ± 0.4
Schisandrin C	5.8 ± 3.2	8.6 ± 0.2	5.1 ± 0.5	5.9 ± 0.1	7.2 ± 0.4	5.4 ± 0.7	6.4 ± 0.2	8.2 ± 0.2	9.0 ± 0.6	7.2 ± 0.6	6.6 ± 0.6	6.2 ± 0.3	9.8 ± 0.9
Angeoyl/tigloyl-gomisin H	36.4 ± 3.6	43.4 ± 2.1	31.6 ± 1.0	24.6 ± 1.3	49.6 ± 3.0	31.4 ± 1.1	33.2 ± 1.2	36.2 ± 3.1	72.1 ± 4.7	35.9 ± 1.4	32.4 ± 1.2	28.5 ± 2.4	47.3 ± 1.1
Angeoyl/tigloylg-omisin Q	76.8 ± 8.1	101.7 ± 2.8	63.7 ± 5.5	55.1 ± 1.8	107.5 ± 4.0	75.5 ± 6.3	81.1 ± 3.1	85.4 ± 5.3	148.1 ± 6.5	83.9 ± 8.2	77.4 ± 6.0	67.8 ± 3.9	115.6 ± 4.5
Schisandrin B	20.7 ± 3.3	30.9 ± 2.9	19.4 ± 3.0	21.8 ± 0.8	26.6 ± 3.1	21.0 ± 0.8	22.1 ± 2.5	25.7 ± 2.8	32.5 ± 2.8	25.4 ± 2.9	23.3 ± 1.1	21.4 ± 4.1	31.9 ± 0.9
Benzoylgomisin P	25.9 ± 3.8	39.8 ± 2.7	24.5 ± 2.0	23.9 ± 1.6	33.8 ± 2.4	27.9 ± 1.0	30.9 ± 1.4	36.0 ± 1.3	46.3 ± 2.8	35.1 ± 2.5	30.8 ± 1.9	28.8 ± 0.8	44.8 ± 1.4
Schisantherin D	8.9 ± 2.5	12.3 ± 0.2	8.1 ± 0.7	9.0 ± 0.6	7.7 ± 1.1	9.4 ± 0.9	11.4 ± 1.5	13.4 ± 0.7	13.7 ± 2.2	11.8 ± 1.7	11.6 ± 1.5	8.5 ± 0.5	11.2 ± 1.1
Total content	366.2 ± 45.1	491.7 ± 23.2*	319.1 ± 32.5	306.1 ± 16.7*	522.9 ± 34.5*	354.5 ± 23.4	369.6 ± 19.0	428.1 ± 21.6*	730.6 ± 53.8*	414.7 ± 26.4*	372.7 ± 28.6*	332.5 ± 17.5*	585.5 ± 29.2*

*p < 0.05 vs control

**Table 2:** Production [mg/100 g DW ± SD] of dibenzocyclooctadiene lignans in agitated microshoot cultures of *S. chinensis* elicited with yeast extract − YeE (*n* = 3)

Lignans	Control	Elicitation scheme [day added/yeast extract ± YeE concentration (mg/l)]											
		0/100	0/1000	0/3000	0/5000	10/100	10/1000	10/3000	10/5000	20/100	20/1000	20/3000	20/5000
Schisandrin	80.5 ± 8.0	73.1 ± 3.7	53.0 ± 4.8	159.3 ± 6.6	175.2 ± 7.7	100.8 ± 3.8	102.4 ± 2.8	75.6 ± 2.7	115.1 ± 5.2	92.6 ± 1.6	126.5 ± 2.5	125.6 ± 2.2	117.3 ± 1.7
Gomisin A	52.8 ± 7.5	58.2 ± 2.5	33.4 ± 0.8	112.0 ± 2.9	87.9 ± 4.2	79.5 ± 4.5	108.5 ± 8.7	89.3 ± 15.6	122.2 ± 7.8	71.2 ± 2.5	139.1 ± 6.1	142.9 ± 6.0	123.6 ± 8.0
Gomisin G	3.9 ± 0.7	3.7 ± 1.0	1.2 ± 0.3	11.8 ± 1.3	11.3 ± 1.4	3.8 ± 0.4	8.3 ± 0.9	5.6 ± 0.6	4.3 ± 1.0	1.6 ± 0.1	2.1 ± 0.1	3.6 ± 1.0	3.2 ± 0.5
Schisantherin A	1.8 ± 0.2	1.0 ± 0.1	1.0 ± 0.1	5.6 ± 0.1	5.6 ± 0.4	1.6 ± 0.1	3.2 ± 0.2	1.6 ± 0.1	2.3 ± 0.1	1.6 ± 0.1	3.0 ± 0.3	4.5 ± 0.1	2.3 ± 0.1
Schisantherin B	10.8 ± 0.2	8.7 ± 0.5	4.9 ± 0.2	14.7 ± 0.8	20.4 ± 0.8	7.1 ± 0.8	15.7 ± 2.2	8.2 ± 0.9	10.9 ± 1.1	7.0 ± 0.1	28.2 ± 0.5	17.6 ± 1.1	14.9 ± 1.1
Schisanthenol	2.5 ± 0.2	2.3 ± 0.4	2.0 ± 0.2	8.8 ± 0.8	5.7 ± 0.3	2.8 ± 0.1	2.9 ± 0.3	5.4 ± 0.2	6.5 ± 0.6	2.9 ± 0.2	2.6 ± 0.1	2.5 ± 0.1	6.8 ± 0.2
Deoxyschisandrin	34.6 ± 3.1	38.2 ± 5.0	26.1 ± 2.3	66.5 ± 4.7	67.4 ± 5.1	47.9 ± 0.9	44.8 ± 3.7	37.2 ± 1.4	58.7 ± 2.6	45.0 ± 1.6	61.5 ± 2.7	67.0 ± 2.0	61.2 ± 4.0
γ-Schisandrin	4.7 ± 0.6	4.4 ± 0.5	3.4 ± 0.3	7.4 ± 0.3	7.7 ± 0.5	5.7 ± 0.3	5.1 ± 0.5	4.7 ± 0.4	7.2 ± 0.4	5.0 ± 0.7	6.4 ± 0.2	6.5 ± 0.3	7.6 ± 0.4
Schisandrin C	5.8 ± 3.2	14.2 ± 1.1	3.4 ± 0.1	10.4 ± 0.5	18.9 ± 1.5	6.5 ± 0.2	13.7 ± 2.6	6.1 ± 0.4	14.5 ± 1.1	5.9 ± 0.9	14.9 ± 1.2	14.7 ± 1.3	9.9 ± 0.8
Angeoyl/tigloyl-gomisin H	36.4 ± 3.6	32.5 ± 1.6	21.7 ± 2.4	64.9 ± 6.2	62.0 ± 3.2	37.8 ± 2.3	34.7 ± 1.4	27.3 ± 2.3	45.5 ± 1.6	35.5 ± 1.4	52.6 ± 0.9	56.1 ± 4.5	47.3 ± 1.1
Angeoyl/tigloyl-gomisin Q	76.8 ± 8.1	79.7 ± 7.9	55.8 ± 5.7	118.5 ± 4.0	142.9 ± 5.6	90.7 ± 0.9	91.2 ± 2.0	72.6 ± 3.8	110.3 ± 4.0	82.4 ± 7.6	135.3 ± 4.9	135.9 ± 3.4	114.4 ± 4.5
Schisandrin B	20.7 ± 3.3	18.6 ± 2.0	15.0 ± 0.3	28.9 ± 0.9	29.3 ± 0.5	22.5 ± 0.3	19.9 ± 1.5	19.1 ± 1.0	28.9 ± 1.0	21.8 ± 0.8	23.7 ± 1.6	27.6 ± 1.3	30.4 ± 1.1
Benzoylgomisin P	25.9 ± 3.8	21.6 ± 0.6	18.5 ± 1.0	40.3 ± 1.7	36.3 ± 1.7	30.4 ± 2.0	23.4 ± 1.7	25.6 ± 2.8	41.0 ± 1.4	29.4 ± 1.3	31.0 ± 2.1	35.8 ± 0.8	43.3 ± 0.7
Schisantherin D	8.9 ± 2.5	11.0 ± 2.2	6.4 ± 0.5	11.2 ± 1.0	8.8 ± 0.6	9.8 ± 1.0	14.0 ± 0.8	9.1 ± 0.7	12.6 ± 0.5	9.8 ± 0.3	13.3 ± 0.6	15.2 ± 0.7	14.5 ± 0.8
Total content	366.2 ± 45.1	367.2 ± 28.9	245.7 ± 18.8*	660.2 ± 31.8*	679.3 ± 33.4*	446.6 ± 17.5*	487.7 ± 29.3*	387.3 ± 32.7*	579.9 ± 28.3*	411.6 ± 19.1*	639.9 ± 23.7*	655.6 ± 24.7*	596.3 ± 24.9*

*p < 0.05 vs control

**Table 3:** Production [mg/100 g DW ± SD] of dibenzocyclooctadiene lignans in agitated microshoot cultures of *S. chinensis* elicited with chitosan − Ch. Control − microshoots without chitosan treatment, S - control microshoots without chitosan treatment, supplemented with neutralized hydrochloric acid solution (used as chitosan solvent). *n* = 3

Lignans	Control	Elicitation scheme [day added/chitosan ± Ch concentration (mg/l)]														
		0/S	0/25	0/50	0/100	0/200	10/S	10/25	10/50	10/100	10/200	20/S	20/25	20/50	20/100	20/200
Schisandrin	80.5 ± 8.0	97.4 ± 10.2	103.7 ± 11.7	116.5 ± 13.0	103.7 ± 3.2	103.3 ± 10.9	99.7 ± 6.4	99.2 ± 8.8	102.1 ± 8.7	114.9 ± 4.6	115.6 ± 13.6	95.2 ± 11.3	93.1 ± 16.7	94.7 ± 9.8	105.6 ± 3.8	92.0 ± 3.7
Gomisin A	52.8 ± 7.5	43.6 ± 6.6	36.0 ± 11.5	51.3 ± 5.3	36.9 ± 4.5	46.3 ± 2.2	44.7 ± 8.4	30.9 ± 12.1	30.3 ± 5.2	44.0 ± 4.8	52.5 ± 15.6	46.4 ± 9.9	24.1 ± 7.0	28.6 ± 2.7	43.6 ± 14.3	32.2 ± 5.1
Gomisin G	3.9 ± 0.7	7.3 ± 0.7	10.0 ± 1.3	10.5 ± 3.3	9.6 ± 0.7	8.6 ± 0.4	6.7 ± 2.4	9.0 ± 1.4	9.9 ± 1.1	10.7 ± 0.6	8.7 ± 1.0	6.0 ± 1.4	8.4 ± 2.5	7.7 ± 0.6	5.8 ± 1.7	5.7 ± 1.1
Schisantherin A	1.8 ± 0.2	3.7 ± 0.2	3.6 ± 0.4	4.3 ± 0.5	3.3 ± 0.2	2.9 ± 0.2	3.8 ± 1.0	3.8 ± 0.4	3.8 ± 0.4	4.4 ± 0.1	5.1 ± 0.4	3.5 ± 0.7	3.7 ± 0.6	3.7 ± 0.4	4.1 ± 0.3	4.1 ± 0.4
Schisantherin B	10.8 ± 0.2	18.6 ± 2.3	17.0 ± 4.4	15.7 ± 2.1	18.0 ± 2.4	16.2 ± 1.4	18.0 ± 2.0	16.3 ± 4.1	17.6 ± 2.2	18.2 ± 1.5	27.9 ± 1.7	19.0 ± 2.6	15.5 ± 5.0	14.6 ± 4.2	15.8 ± 2.9	17.4 ± 2.2
Schisanthenol	2.5 ± 0.2	4.4 ± 0.3	5.9 ± 0.5	6.1 ± 0.6	5.3 ± 0.3	4.6 ± 0.1	3.8 ± 0.9	4.1 ± 0.9	4.3 ± 0.1	5.0 ± 0.4	4.7 ± 0.8	4.5 ± 1.0	4.8 ± 0.5	4.1 ± 0.7	4.2 ± 0.4	4.0 ± 0.1
Deoxyschisandrin	34.6 ± 3.1	29.2 ± 3.7	32.4 ± 5.5	37.1 ± 4.9	29.9 ± 2.0	30.3 ± 1.2	29.8 ± 2.2	30.9 ± 5.6	28.1 ± 1.3	31.9 ± 2.4	34.3 ± 7.4	27.3 ± 2.7	21.6 ± 6.5	15.7 ± 5.8	33.0 ± 5.3	21.6 ± 1.3
γ-Schisandrin	4.7 ± 0.6	6.0 ± 1.0	6.2 ± 0.7	8.6 ± 0.9	5.6 ± 1.0	6.4 ± 0.3	5.2 ± 0.6	6.3 ± 0.9	6.1 ± 0.4	6.5 ± 0.3	6.6 ± 1.0	7.1 ± 1.0	4.9 ± 1.0	5.2 ± 0.7	6.2 ± 0.8	4.9 ± 0.4
Schisandrin C	5.8 ± 3.2	1.0 ± 0.3	1.9 ± 0.3	4.0 ± 1.6	1.8 ± 0.7	2.3 ± 0.5	1.1 ± 0.2	1.3 ± 0.4	1.5 ± 0.2	3.3 ± 1.6	3.8 ± 1.2	0.8 ± 0.4	0.5 ± 0.8	0.8 ± 0.9	1.5 ± 0.7	0.4 ± 0.2
Angeoyl/tigloyl-gomisin H	36.4 ± 3.6	49.8 ± 2.4	53.1 ± 6.8	61.0 ± 13.4	47.1 ± 4.1	51.8 ± 6.7	48.1 ± 2.4	49.7 ± 6.3	49.1 ± 3.2	57.3 ± 2.6	61.7 ± 8.3	59.4 ± 5.3	43.3 ± 8.3	45.8 ± 5.3	51.8 ± 7.8	46.3 ± 3.2
Angeoyl/tigloyl-gomisin Q	76.8 ± 8.1	71.5 ± 5.2	102.7 ± 9.8	113.2 ± 4.1	103.4 ± 12.4	102.2 ± 3.1	70.8 ± 7.9	93.2 ± 9.5	92.2 ± 4.6	107.8 ± 5.2	112.9 ± 11.2	68.3 ± 7.0	87.4 ± 6.5	91.0 ± 10.2	96.9 ± 8.1	87.2 ± 4.6
Schisandrin B	20.7 ± 3.3	25.1 ± 2.4	24.8 ± 2.3	33.0 ± 3.7	23.9 ± 0.4	25.6 ± 0.5	16.5 ± 4.3	24.6 ± 2.4	24.6 ± 0.6	14.5 ± 0.2	25.7 ± 3.0	30.1 ± 4.1	23.1 ± 2.8	22.0 ± 2.0	25.1 ± 2.7	20.9 ± 0.6
Benzoylgomisin P	25.9 ± 3.8	32.3 ± 2.3	31.5 ± 3.1	34.5 ± 2.0	32.1 ± 3.1	31.1 ± 2.9	38.3 ± 5.2	30.9 ± 2.6	30.9 ± 0.6	34.1 ± 2.3	30.3 ± 3.5	32.5 ± 3.9	27.3 ± 4.6	26.8 ± 2.7	30.7 ± 3.9	24.8 ± 0.6
Schisantherin D	8.9 ± 2.5	9.5 ± 1.2	10.4 ± 1.0	12.4 ± 0.7	10.7 ± 0.3	11.1 ± 0.4	9.3 ± 1.4	10.5 ± 0.5	10.4 ± 0.4	10.9 ± 0.6	12.0 ± 0.9	9.4 ± 1.0	10.1 ± 0.7	10.2 ± 0.4	11.0 ± 1.1	9.7 ± 0.4
Total content	366.2 ± 45.1	399.1 ± 38.6*	439.1 ± 59.5*	508.1 ± 55.9*	431.2 ± 35.2*	442.6 ± 31.9*	395.9 ± 45.0*	410.8 ± 55.9*	410.7 ± 28.9*	463.6 ± 27.2*	501.9 ± 69.5*	409.5 ± 52.0*	367.8 ± 63.5	370.8 ± 46.2	435.1 ± 53.7*	371.2 ± 23.9

*p < 0.05 vs control

**Table 4:** Production [mg/100 g DW ± SD] of dibenzocyclooctadiene lignans in agitated microshoot cultures of *S. chinensis* and maintained in Plantform temporary immersion system elicited with 1000 mg/l of yeast extract (YeE) on the 20th day. *n* = 4

Lignans	Agitated microshoots		Plantform bioreactor	
	Control	YeE elicitation	Control	YeE elicitation
Schisandrin	80.5 ± 8.0	126.5 ± 2.5	128.7 ± 17.8	186.8 ± 9.7
Gomisin A	52.8 ± 7.5	139.1 ± 6.1	55.4 ± 17.6	97.2 ± 8.5
Gomisin G	3.9 ± 0.7	2.1 ± 0.1	6.1 ± 0.8	11.5 ± 1.0
Schisantherin A	1.8 ± 0.2	3.0 ± 0.3	2.6 ± 0.2	4.8 ± 0.3
Schisantherin B	10.8 ± 0.2	28.2 ± 0.5	14.6 ± 2.1	26.0 ± 2.1
Schisanthenol	2.5 ± 0.2	2.6 ± 0.1	2.1 ± 0.2	3.7 ± 0.2
Deoxyschisandrin	34.6 ± 3.1	61.5 ± 2.7	62.5 ± 14.6	100.0 ± 8.2
γ-Schisandrin	4.7 ± 0.6	6.4 ± 0.2	7.2 ± 0.3	13.6 ± 1.6
Schisandrin C	5.8 ± 3.2	14.9 ± 1.2	2.2 ± 1.6	8.3 ± 0.6
Angeoyl/tigloyl-gomisin H	36.4 ± 3.6	52.6 ± 0.9	35.0 ± 10.6	85.2 ± 5.5
Angeoyl/tigloyl-gomisin Q	76.8 ± 8.1	135.3 ± 4.9	104.4 ± 3.4	183.4 ± 11.9
Schisandrin B	20.7 ± 3.3	23.7 ± 1.6	24.5 ± 1.4	41.8 ± 2.3
Benzoylgomisin P	25.9 ± 3.8	31.0 ± 2.1	32.6 ± 0.7	56.5 ± 3.4
Schisantherin D	8.9 ± 2.5	13.3 ± 0.6	11.0 ± 0.5	12.8 ± 0.5
Total content	366.2 ± 45.1	639.9 ± 23.8*	488.8 ± 71.9	831.6 ± 55.8*

*p < 0.05 vs control

Cadmium ions proved to be the most effective at 1000 μM when applied on the first, 10th, or 20th day of the experiment, with the total contents of *Schisandra* lignans equal to 522.9, 730.6, and 585.5 mg/100 g DW, respectively (Table 1). Correspondingly, these amounts were 1.4, 2.0, and 1.6 times higher than in the control samples. The contents of the main *Schisandra* lignans, schisandrin and gomisin A, in the extracts from the biomass collected after the elicitation with 1000 μM on the tenth day, were equal to 183.6 mg/100 g DW and 115.9 mg/100 g DW and were up to 2.4 and 2 times higher than in the control samples, respectively. The rest of the lower concentrations of CdCl_2_ added on the first, 10th and 20th days of microshoot growth periods slightly influenced on the lignan production.

The experiments involving biotic elicitor, YeE, supplemented in concentrations: 100, 1000, 3000, and 5000 mg/l on the first, 10th, or 20th day of the experiment, as shown in Table 2, caused up to 1.85-time increase in the total production of *Schisandra* lignans, in the agitated microshoots. The highest total amounts of lignans were estimated in the extracts from microshoots, elicited with 3000 mg/l of YeE (660.2 mg/100 g DW) and 5000 mg/l of YeE (679.3 mg/100 g DW) on the first day, 5000 mg/l (572.7 mg/100 g DW) on the tenth day, and 1000 mg/l (639.9 mg/100 g DW), 3000 mg/l (655.6 mg/100 g DW), and 5000 mg/l (596.3 mg/100 g DW) on the 20th day of the experiment. The highest amounts of the main, individual compounds were confirmed as follows: schisandrin—175.2 mg/100 g DW (5000 mg/l YeE on the 1st day), and gomisin A—139.1 mg/100 g DW (1000 mg/l YeE on the 20th day of experiment). The most effective for the total and individual lignan production as well as the biomass increments was the elicitation scheme with 1000 mg/l YeE on the 20th day of experiment (Fig. 2 and Table 2).

Elicitation with the second biotic elicitor, Ch (chitosan), in all the tested solutions on the 20th day of the growth period only marginally influenced the production of *Schisandra* lignans (Table 3). The most favorable results were obtained for the microshoots elicited on the first and the tenth day. In these samples, the total amounts of lignans were quite similar, ranging from 410.7 mg/100 g DW (100 mg/l Ch on the tenth day) to 508.1 mg/100 g DW (50 mg/l Ch on the first day). The maximal total content of lignans was ca. 1.3 times higher than in the control sample. The maximal contents of main *Schisandra* lignans amounted ca. 115 mg/100 g DW for schisandrin, 52 mg/100 g DW for gomisin A, and 35 mg/100 g DW for deoxyschisandrin and were detected after elicitation with 50 mg/l on the first day and 200 mg/l on the tenth day of experiment.

### Scaling up of the elicitation process in the temporary immersion bioreactors

For the bioreactor experiment was chosen the most effective elicitation protocol, selected by the review of experiments conducted on the agitated cultures (Figs. 1, 2, and 3, Tables 1, 2, and 3). The employed elicitation scheme involved YeE, supplemented at 1000 mg/l, on the 20th day of the growth period. The choice was based on the good growth parameters, high lignan content (Fig. 2, Table 2), and nontoxicity of YeE.

**Fig. 4:**
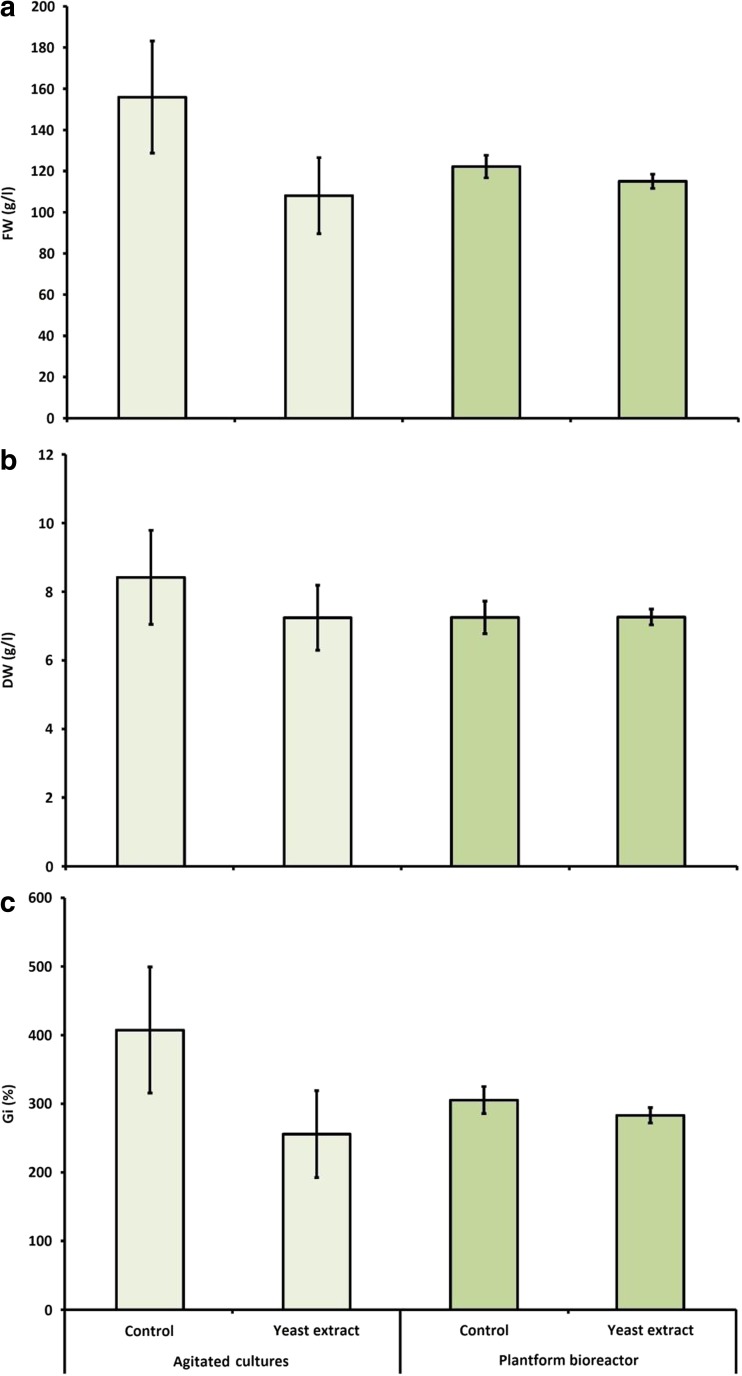
The effect of yeast extract (added at 1000 mg/l on day 20) on biomass growth in agitated and bioreactor-grown shoot cultures of *S. chinensis*. **a** Fresh weight (FW). **b** Dry weight (DW). **c** Growth index (Gi). The cultures were grown for 30 days

The growth parameters of the elicited microshoots grown in a bioreactor were not considerably changed in relation to the control experiments. In both cases, the Gi factor oscillated about 300% (Fig. 4). There were no differences in the morphology of the elicited and non-elicited shoots.

The total obtained amount (831.6 mg/100 g DW) of *Schisandra* lignans in the elicited microshoots improved 1.7 times in comparison with the untreated biomass, maintained in bioreactor (488.8 mg/100 g DW) (Table 4). The total amounts of lignans in elicited bioreactor culture were the highest in the presented series of experiments. In the extracts from elicited microshoots cultured in Plantform bioreactor, high amounts of the main lignans: schisandrin—186.8 mg/100 g DW, gomisin A—97.2 mg/100 g DW, and deoxyschisandrin—100.0 mg/100 g DW, were estimated.

## Discussion

The preliminary elicitation experiments were based on the results of the previous studies on lignan-producing plant in vitro cultures. These reports indicated that methyl jasmonate (MeJa), applied at 50–400 μM and not earlier than 9 days before the end of an experiment, noticeably increased the accumulation of aryltetralin lignans (Bahabadi et al. 2011; Bhattacharyya et al. 2012; Wawrosch et al. 2014). In the course of our experiments on *S. chinensis* microshoots, the biomass was elicited with MeJa at 50, 100, and 200 μM on the 23rd and/or the 27th day of the growth cycle and collected on the 30th day (Figure S3, Table S2). Moreover, the experiments included cadmium chloride (CdCl_2_) (Figure S4, Table S3), which was previously shown to increase the accumulation of biologically active lignans (phyllanthin and hypophyllanthin) in *Phyllanthus amarus* (Rai et al. 2005), and dimethylsulfoxide (DMSO) (Figure S2, Table S1), demonstrated to act as both elicitor of secondary metabolism (Mannan et al. 2010) and an effective permeabilizing agent (Luczkiewicz and Kokotkiewicz 2012; Jaremicz et al. 2014). CdCl_2_ was applied at 2.5, 5, 10, and 20 mM, and DMSO was added at 0.2, 2, 4, and 8% *v/v*. As in the case of MeJa, both agents were added on the 23th or the 27th day and the experiment was run for 30 days.

Owing to the results of the preliminary studies, further elicitation experiments were designed. These were aimed at examining the influence of varying concentrations of the elicitors, added on different phases of the growth period (Figs. 1, 2, and 3 and Tables 1, 2, and 3), on shoot growth and lignan accumulation. The experiments included CdCl_2_ (the most effective elicitor in preliminary work), whereas MeJa and DMSO were excluded from the studies. Besides CdCl_2_, two biotic elicitors were used in the experiment: yeast extract (YeE) and chitosan (Ch). Based on the other studies, both YeE and Ch were demonstrated to stimulate aryltetralin lignan accumulation in plant cell and organ cultures (Esmaeilzadeh Bahabadi et al. 2014; Wawrosch et al. 2014; Malik et al. 2014).

The amounts of the main *Schisandra* lignans were estimated in the methanolic extracts from the elicited microshoots, collected after 30-day growth periods. In general, the elicitation procedures did not affect the qualitative composition of the lignan set present in *S. chinensis* microshoots. Irrespectively of the applied elicitation strategy, only the traces of the studied lignans were detected in the experimental media samples.

The conducted experiments confirm that elicitation with CdCl_2_ is an effective means of improving dibenzocyclooctadiene lignan production in *S. chinensis* cultures. The results also show that CdCl_2_ concentration can be reduced up to 20 times while retaining the elicitor’s efficacy (Table 1 and Table S3). So far, there have been relatively few reports on the effects of cadmium ions on the plant secondary metabolism. Besides boosting lignan accumulation in *Phyllanthus amarus* (Rai et al. 2005), they also proved to be effective (best results at 2.0 mM) at stimulating the biosynthesis of gymnemic acid (saponin) accumulation in *Gymnema sylvestre* suspension cultures (Ch and Rao 2012), tanshinone - diterpene (best results at 25 μM) in suspension cultures of *Salvia miltiorrhiza* (Zhao et al. 2010), and anthracene derivatives (at 10 μM) in the suspension culture of *Rheum palmatum* (Kasparová and Siatka 2004). The results of the abovementioned studies, as well as the present work, indicate that CdCl_2_ seems to be effective as an elicitor in a wide concentration range. However, its use should be limited due to an environmental burden as well as the need to remove cadmium ion residuals from the harvested biomass.

Given the toxicity of cadmium salts, the efforts were made to replace CdCl_2_ with safer elicitors. In the current work, the effects of two biotic elicitors (Ch and YeE) on the production of *Schisandra* lignans have been examined for the first time.

The most plausible results were obtained for the elicitation conducted on the 20th day of experiment. Interestingly, the selected YeE (yeast extract) elicitation schemes noticeably increased the accumulation of gomisin A (Table 2) whose concentrations exceeded schisandrin content (major lignan content in majority of the samples). At 1000 mg/l added on the 20th day, YeE stimulated the accumulation of lignans while not negatively affecting biomass growth. Therefore, the abovementioned elicitation protocol was applied for bioreactor-grown *S. chinensis* microshoots.

YeE and Ch are known as effective and relatively cheap elicitors, commonly employed for enhancing secondary metabolism in plant in vitro cultures (Ramirez-Estrada et al. 2016). For instance, YeE stimulated the accumulation of andrographolide—specific diterpene lactone—responsible for pharmacological action of *Andrographis paniculata*, in suspension cultures of the aforementioned plant species (8.8-fold increase as compared with untreated cells) (Gandi et al. 2012). More importantly, in several studies, YeE has been demonstrated to be the most effective agent that increased lignan accumulation. For example, YeE considerably increased the production of lariciresinol-type lignan—leolignin—in the ‘hairy root’ cultures of *Leontopodium nivale* ssp. *alpinum*, providing the amounts of lignans comparable to those found in roots of the parent plants (Wawrosch et al. 2014). At 1000 mg/l, YeE was also reported to boost the biosynthesis of the specific neolignans—8,4′-oxynorneolignans—in *Echinacea purpurea* cell cultures (Li and Barz 2005). Moreover, it the efficacy of YeE at stimulating the production of flavonolignans (including silychristin, silydianin, silybin, and isosilybin) was demonstrated in cell suspension cultures of *Silybum marianum* (Firouzi et al. 2013).

Among the elicitors employed in the present study, the second biotic elicitor, chitosan (Ch), proved to be the least effective at improving lignan accumulation. This result is in agreement with the previous reports. For instance, Ch proved to be one of the less effective elicitors applied in order to enhance the accumulation of podophyllotoxin and the related lignan constituents in *Linum album* cell suspension cultures (Bahabadi et al. 2011).

Ch was found to be a good elicitor in the experiment with *Withania somnifera* (Sivanandhan et al. 2014) suspension cultures. Ch was selected as the most favoring for the accumulation of withanolides in the agitated flasks, as well as in the bioreactor cultures; the amounts were 2.1 and 1.7 times higher than in the unelicited biomass extracts.

On the basis of our previous experiments, on the influence of different types of bioreactors on the growth parameters, and production of lignans in *S. chinensis* microshoots (Szopa et al. 2017b), we selected the Plantform temporary immersion system, which provided the highest lignan content, as the most favorable one, for the current work. As demonstrated in the previous reports (Pérez-Alonso et al. 2009; Georgiev et al. 2014; Ptak et al. 2017), the temporary immersion systems are also suitable for the elicitation experiments.

The results obtained from the bioreactor experiment offer the possibility to advance the production of *Schisandra* lignans, based on increasing their content in view of an elicitation method. Correspondingly, there are the attempts to boost the production of taxuyunnanine C, the taxane precursor, which is the plant metabolite of the great anticancer value, in suspension cultures of *Taxus chinensis* by maintaining the culture in airlift bioreactor, elicited with MeJa (Dong and Zhong 2002). Promising, though, are also the results in increasing ginsenoside production, a metabolite of an outstanding biological activity, which were established after MeJa elicitation of cell suspension cultures of *Panax ginseng*, in 5-liter balloon-type bubble bioreactors (Thanh et al. 2005).

**Fig. 5:**
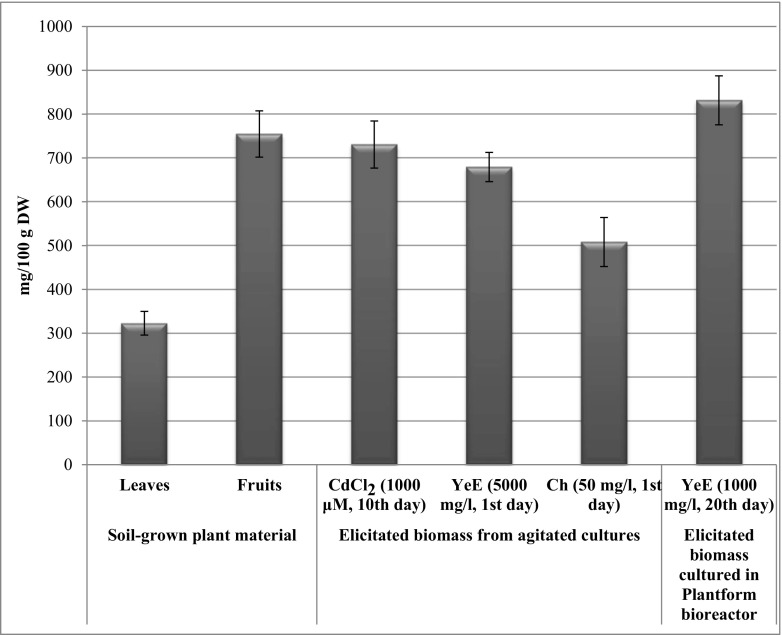
Comparison of reached maximal total contents [mg/100 g DW ± SD] of dibenzocyclooctadine lignans in agitated and bioreactor microshoot cultures of *S. chinensis*, obtained after optimization of the elicitation process, with their contents in reference plant material—leaves and fruits of soil-grown plant (Szopa et al. 2016b)

The elicitation experiments on *S. chinensis* microshoots provided promising results in terms of improving dibenzocyclooctadiene lignan accumulation. The maximal total contents of *Schisandra* lignans, estimated in the elicited cultures, were higher than in the leaves of the parent plant and comparable (or even higher) to the pharmacopoeial plant raw material—*Schisandra* fruits (Fig. 5). The highest amount of the lignans produced by the biomass elicited with 1000 mg/l YeE on the 20th day in Plantform bioreactor was 10% higher than in fruits and 39% higher than in the leaves of the parent plant (Fig. 5) (Szopa et al. 2016b). The successful transfer of the elicitation experiment from the agitated cultures to the bioreactor scale offers the possibility to advance the production of *Schisandra* lignans and creates prospects for practical applications of the presented results.

## Electronic supplementary material

ESM 1 (DOCX 783 kb)
