# Supplementary material for: Improved production of dibenzocyclooctadiene lignans in the elicited microshoot cultures of Schisandra chinensis (Chinese magnolia vine)
Source: Appl Microbiol Biotechnol. 2017 Nov 27;102(2):945–59. doi: 10.1007/s00253-017-8640-7 (PMC5756551; doi:10.1007/s00253-017-8640-7)
Supplement: Supplementary file 1 — (DOCX 783 kb) [file 253_2017_8640_MOESM1_ESM.docx]

**Applied Microbiology and Biotechnology**

original article

**Improved production of dibenzocyclooctadiene lignans in the elicited microshoot cultures of *Schisandra chinensis* (Chinese magnolia vine)**

Szopa Agnieszka*^1^, Kokotkiewicz Adam^2^, Król Agata^2^, Luczkiewicz Maria^2^, Ekiert Halina^1^

*^1^ Chair and Department of Pharmaceutical Botany, Jagiellonian University, Collegium Medicum, ul. Medyczna 9,
30-688 Kraków, Poland,*

*^2^ Chair and Department of Pharmacognosy, Faculty of Pharmacy, Medical University of Gdansk,
al. gen. J. Hallera 107, 80-416 Gdańsk, Poland*

*Corresponding author:

phone +48 12 620 54 30, fax +48 620 54 40, e-mail: a.szopa@uj.edu.pl

| Deoxyschisandrin   | Gomisin A   | Schisandrin   |
| --- | --- | --- |
| Schisandrin B   | Schisandrin C   | γ-Schisandrin   |
| Schisanthenol   | Schisantherin A   | Schisantherin B   |

**Figure S1**. The chemical structures of studied dibenzocyclooctadiene lignans (acc Szopa et al. 2017).


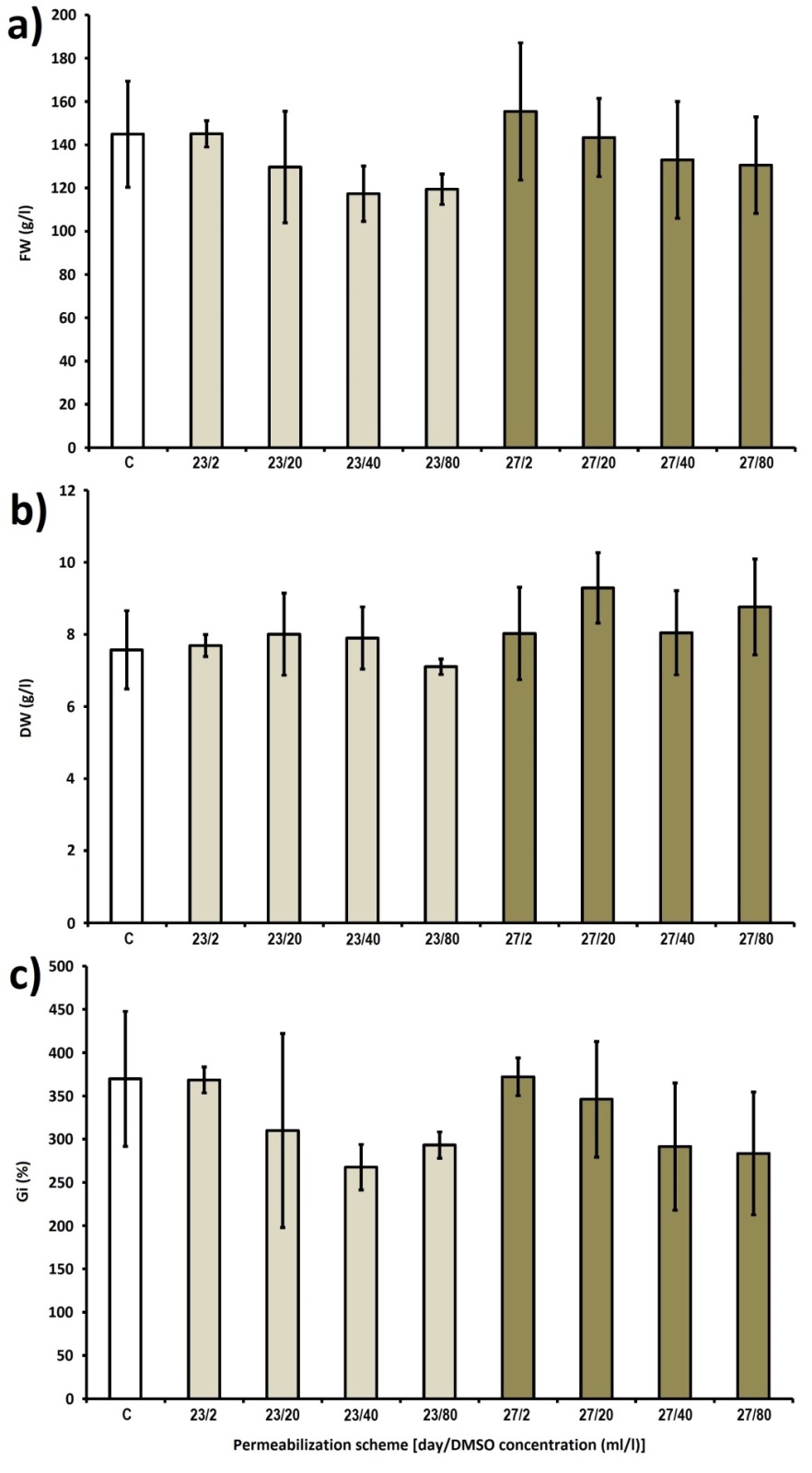


**Figure S2.** The effect of dimethyl sulfoxide (DMSO)* on biomass growth in agitated shoot cultures of *S. chinensis*†: **a)** fresh weight (FW), **b)** dry weight (DW), **c)** growth index (Gi)‡. Symbols used: **C -** control group (shoots grown without DMSO). All cultures were grown for 30 days

* molecular biology grade (Sigma-Aldrich), sterile-filtered prior to use (2.0 ml per flask)

† medium composition and growth room conditions as described by Szopa et al. (2016). Cultures grown in 125 ml shake flasks with silicone sponge closures [1.5 g shoots/flask/50 ml medium; SHKA 2000 rotary shaker (Barnstead International, Dubuque, US-IA), 125 rpm)]

‡ calculated according to the formula: Gi = [(FW1 – FW0)/FW1] x 100 where FW1 is the fresh weight of shoots at the end
of experiment and FW0 is the fresh weight of the inoculum

**
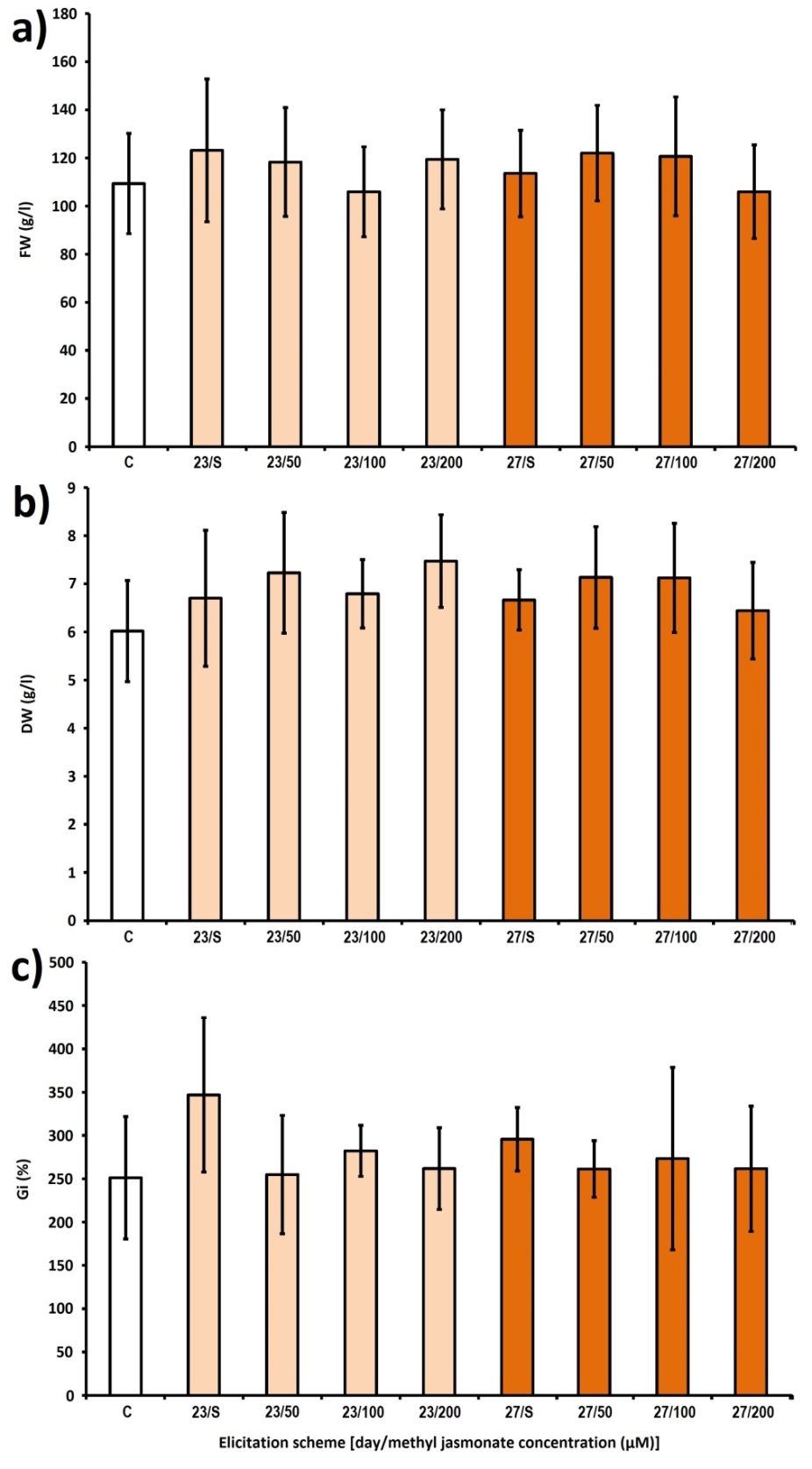
**

**Figure S3**. The effect of methyl jasmonate* on biomass growth in agitated shoot cultures of *S. chinensis*†: **a)** fresh weight (FW), **b)** dry weight (DW), **c)** growth index (Gi)‡. Symbols used: **C** - control group (shoots grown without MeJa/DMSO solution), **S -** shoots supplemented with DMSO only (0.1 ml per flask, final concentration in the growth medium: 2 ml/l). All cultures were grown for 30 days

* 95% grade (Sigma-Aldrich, St. Louis, US-MO) added as sterile-filtered (0.2 μm nylon filter) DMSO stock solutions (0.1 ml per flask, final DMSO concentration in the growth medium: 2 ml/l)

† medium composition and growth room conditions as described by Szopa et al. (2016). Cultures grown in 125 ml shake flasks with silicone sponge closures [1.5 g shoots/flask/50 ml medium; SHKA 2000 rotary shaker (Barnstead International, Dubuque, US-IA), 125 rpm)]

‡ calculated according to the formula: Gi = [(FW1 – FW0)/FW1] x 100 where FW1 is the fresh weight of shoots at the end
of experiment and FW0 is the fresh weight of the inoculum


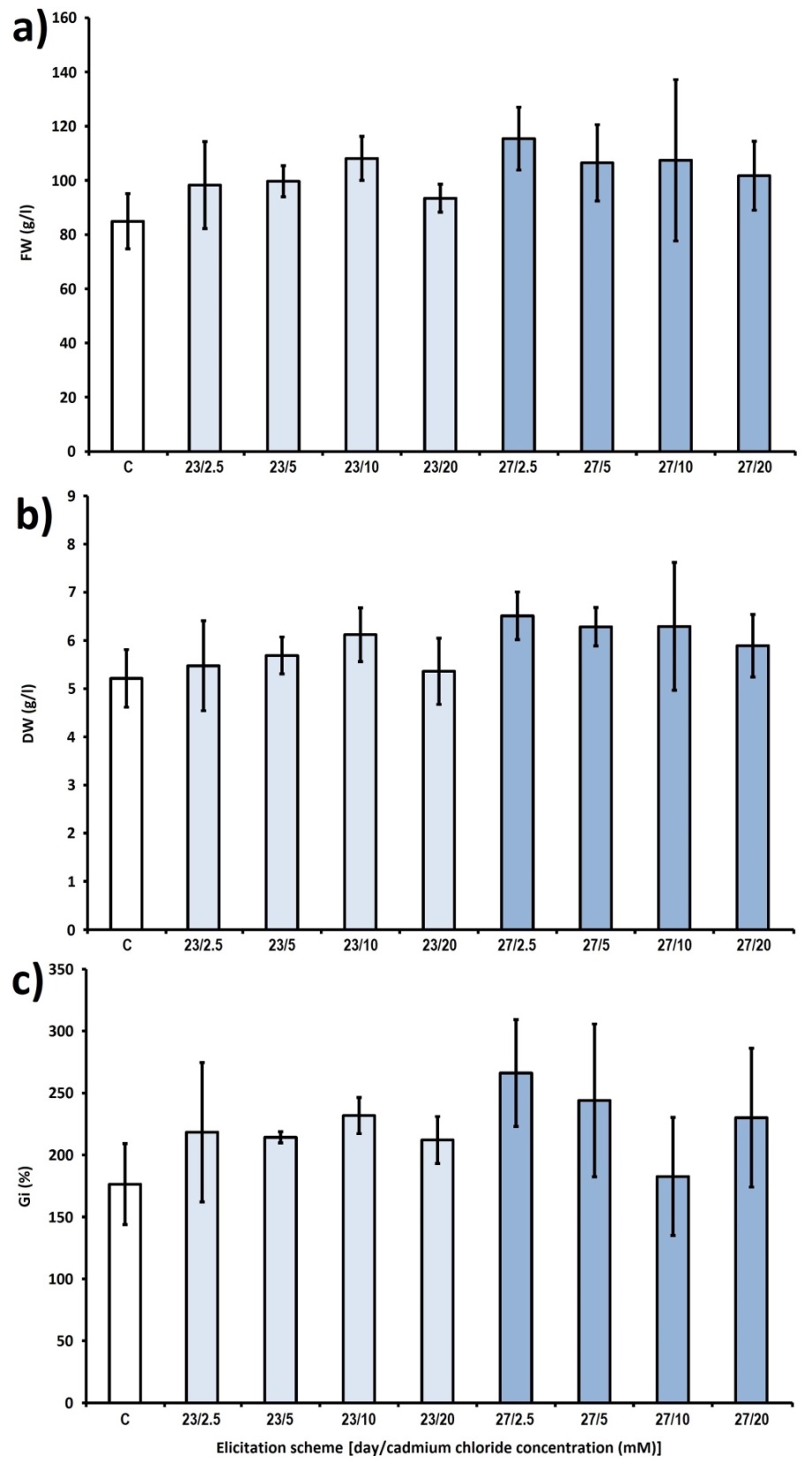


**Figure S4.** The effect of cadmium chloride* on biomass growth in agitated shoot cultures of *S. chinensis*†: **a)** fresh weight (FW), **b)** dry weight (DW), **c)** growth index (Gi)‡. Symbols used: **C -** control group (shoots grown without cadmium chloride). All cultures were grown for 30 days

* reagent grade (POCH, Gliwice, Poland), added as steam-sterilized (120^o^C, 1 bar, 20 min) aqueous stock solutions (2.0 ml per flask)

† medium composition and growth room conditions as described by Szopa et al. (2016). Cultures grown in 125 ml shake flasks with silicone sponge closures [1.5 g shoots/flask/50 ml medium; SHKA 2000 rotary shaker (Barnstead International, Dubuque, US-IA), 125 rpm)]

‡ calculated according to the formula: Gi = [(FW1 – FW0)/FW1] x 100 where FW1 is the fresh weight of shoots at the end
of experiment and FW0 is the fresh weight of the inoculum


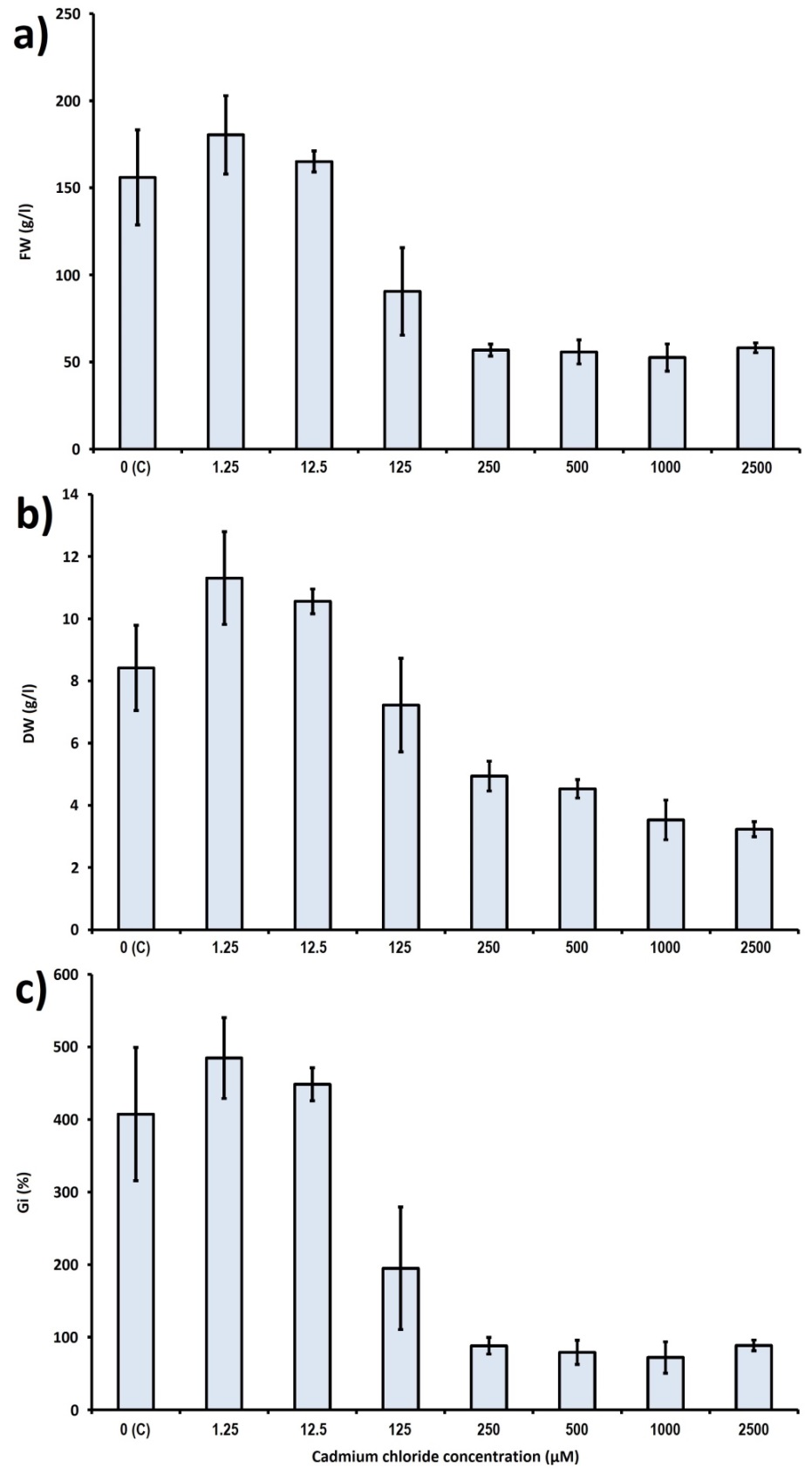


**Figure S5**. The effect of cadmium chloride*, added on day 0, on biomass growth in agitated shoot cultures of *S. chinensis*†: **a)** fresh weight (FW), **b)** dry weight (DW), **c)** growth index (Gi)‡. Symbols used: **C -** control group (shoots grown without cadmium chloride). All cultures were grown for 30 days

* reagent grade (POCH, Gliwice, Poland), added as steam-sterilized (120^o^C, 1 bar, 20 min) aqueous stock solutions (2.0 ml per flask)

† medium composition and growth room conditions as described by Szopa et al. (2016). Cultures grown in 125 ml shake flasks with silicone sponge closures [1.5 g shoots/flask/50 ml medium; SHKA 2000 rotary shaker (Barnstead International, Dubuque, US-IA), 125 rpm)]

‡ calculated according to the formula: Gi = [(FW1 – FW0)/FW1] x 100 where FW1 is the fresh weight of shoots at the end
of experiment and FW0 is the fresh weight of the inoculum

**Table S1.** Production [mg/100 g DW ± SD] of dibenzocyclooctadiene lignans in agitated microshoot cultures of *S. chinensis* treated on 23^rd^ and 27^th^ days of 30 days growth cycles with different concentrations of permeabilizing agent - dimethyl sulfoxide (DMSO) [ml per litr of medium]. Control – agitated microshoots without DMSO treatment. n ± 3, **p*<0.05 *vs* Control.

| **Lignans** | **Control** | **Permeabilization scheme [day added /DMSO concentration (ml/l)]** | | | | | | | |
| --- | --- | --- | --- | --- | --- | --- | --- | --- | --- |
|  |  | **23/2** | **23/20** | **23/40** | **23/80** | **27/2** | **27/20** | **27/40** | **27/80** |
| **Schisandrin** | 80.5  ± 8.0 | 96.4  ± 2.3 | 78.3  ± 2.7 | 66.7  ± 2.8 | 82.2  ± 9.3 | 79.9  ± 2.3 | 81.6  ± 2.2 | 53.1  ± 4.0 | 86.0  ± 6.3 |
| **Gomisin A** | 52.8  ± 7.5 | 58.1  ± 5.7 | 48.7  ± 2.1 | 40.5  ± 3.2 | 40.7  ± 6.5 | 47.5  ± 5.7 | 48.9  ± 2.2 | 29.9  ± 0.5 | 45.1  ± 8.4 |
| **Gomisin G** | 3.9  ± 0.7 | 2.1  ± 0.1 | 1.5  ± 0.1 | 1.2  ± 0.1 | 2.6  ± 0.5 | 2.2  ± 0.1 | 1.6  ± 0.1 | 0.9  ± 0.1 | 1.7  ± 0.3 |
| **Schisantherin A** | 1.8  ± 0.2 | 2.3  ± 0.1 | 2.0  ± 0.1 | 1.5  ± 0.1 | 1.5  ± 0.4 | 1.7  ± 0.1 | 2.4  ± 0.1 | 1.7  ± 0.1 | 1.8  ± 0.4 |
| **Schisantherin B** | 10.8  ± 0.2 | 7.3  ± 0.3 | 6.3  ± 0.2 | 5.6  ± 0.4 | 6.0  ± 0.6 | 6.1  ± 0.3 | 7.6  ± 0.2 | 3.9  ± 0.2 | 5.7  ± 0.5 |
| **Schisanthenol** | 2.5  ± 0.2 | 0.8  ± 0.1 | 0.6  ± 0.1 | 0.4  ± 0.1 | 0.7  ± 0.1 | 0.7  ± 0.1 | 1.0  ± 0.1 | 0.3  ± 0.1 | 0.8  ± 0.1 |
| **Deoxyschisandrin** | 34.6  ± 3.1 | 60.8  ± 4.4 | 51.9  ± 3.1 | 46.0  ± 1.8 | 53.9  ± 2.1 | 50.8  ± 4.4 | 51.1  ± 1.3 | 34.1  ± 0.8 | 50.1  ± 5.6 |
| **γ-Schisandrin** | 4.7  ± 0.6 | 14.3  ± 1.0 | 12.6  ± 0.8 | 10.8  ± 0.7 | 13.1  ± 0.8 | 12.5  ± 1.0 | 13.6  ± 0.5 | 7.4  ± 0.8 | 13.5  ± 0.9 |
| **Schisandrin C** | 5.8  ± 3.2 | 4.4  ± 0.1 | 3.0  ± 0.4 | 2.7  ± 0.1 | 2.8  ± 0.4 | 3.3  ± 0.1 | 3.4  ± 0.1 | 2.2  ± 0.1 | 3.6  ± 0.8 |
| **Angeoyl-/tigloylgomisin H** | 36.4  ± 3.6 | 49.7  ± 2.3 | 38.5  ± 0.8 | 31.7  ± 1.8 | 45.2  ± 4.7 | 39.2  ± 2.3 | 41.9  ± 1.9 | 25.3  ± 1.0 | 37.2  ± 1.3 |
| **Angeoyl-/tigloylgomisin Q** | 76.8  ± 8.1 | 92.5  ± 2.3 | 73.1  ± 2.8 | 59.0  ± 3.6 | 76.9  ± 9.1 | 77.6  ± 2.3 | 78.7  ± 2.5 | 46.4  ± 2.1 | 71.2  ± 12.1 |
| **Schisandrin B** | 20.7  ± 3.3 | 25.6  ± 2.0 | 20.1  ± 0.8 | 17.6  ± 1.4 | 19.9  ± 4.3 | 21.3  ± 2.0 | 21.5  ± 2.0 | 14.0  ± 1.3 | 23.5  ± 0.5 |
| **Benzoylgomisin P** | 25.9  ± 3.8 | 31.0  ± 2.1 | 26.9  ± 1.2 | 23.6  ± 0.8 | 23.1  ± 2.0 | 26.6  ± 2.1 | 25.6  ± 0.8 | 16.2  ± 1.4 | 26.1  ± 1.6 |
| **Schisantherin D** | 8.9  ± 2.5 | 6.3  ± 2.5 | 7.0  ± 0.2 | 5.6  ± 0.2 | 5.4  ± 0.3 | 9.0  ± 2.5 | 5.5  ± 0.1 | 4.8  ± 0.1 | 6.2  ± 0.2 |
| **Total content** | **366.2**  **± 45.1** | **451.6**  **± 25.21*** | **370.3**  **± 15.4** | **312.9**  **± 17.07*** | **373.7**  **± 40.9** | **378.5**  **± 25.21*** | **384.2**  **± 13.93*** | **239.9**  **± 12.38*** | **372.6**  **± 38.8** |

**Table S2.** Production [mg/100 g DW ± SD] of dibenzocyclooctadiene lignans in agitated microshoot cultures of *S. chinensis* elicitated on 23^rd^ and 27^th^ days of 30 days growth cycles with different concentrations of methyl jasmonate – MeJa (µM). Control - microshoots without MeJa/DMSO solution, D - miroshoots supplemented with DMSO only - 0.1 ml per flask, final concentration in the growth medium: 2 ml/l (DMSO was MeJa solvent). n ± 3, **p*<0.05 *vs* Control.

| **Lignans** | **Control** | **Elicitation scheme [day added /methyl jasmonate – MeJa concentration (µM)]** | | | | | | | |
| --- | --- | --- | --- | --- | --- | --- | --- | --- | --- |
|  |  | **23/D** | **23/50** | **23/100** | **23/200** | **27/D** | **27/50** | **27/100** | **27/200** |
| **Schisandrin** | 80.5  ± 8.0 | 68.0  ± 5.2 | 88.4  ± 8.1 | 70.9  ± 9.6 | 68.7  ± 7.5 | 90.7  ± 9.2 | 66.0  ± 6.5 | 87.7  ± 8.3 | 71.2  ± 7.8 |
| **Gomisin A** | 52.8  ± 7.5 | 33.6  ± 2.4 | 47.2  ± 5.1 | 34.5  ± 2.5 | 34.4  ± 5.6 | 42.6  ± 3.7 | 30.4  ± 3.5 | 44.9  ± 4.0 | 35.4  ± 4.6 |
| **Gomisin G** | 3.9  ± 0.7 | 1.6  ± 0.6 | 1.7  ± 0.1 | 1.5  ± 0.1 | 1.3  ± 0.1 | 2.0  ± 0.7 | 1.5  ± 0.1 | 1.7  ± 0.1 | 1.5  ± 0.1 |
| **Schisantherin A** | 1.8  ± 0.2 | 1.4  ± 0.2 | 2.0  ± 0.1 | 1.3  ± 0.1 | 1.5  ± 0.1 | 1.7  ± 0.2 | 1.1  ± 0.1 | 1.7  ± 0.1 | 1.5  ± 0.1 |
| **Schisantherin B** | 10.8  ± 0.2 | 4.8  ± 1.0 | 7.0  ± 0.2 | 4.6  ± 0.1 | 4.7  ± 0.1 | 6.2  ± 0.9 | 4.2  ± 0.1 | 5.8  ± 0.2 | 4.9  ± 0.1 |
| **Schisanthenol** | 2.5  ± 0.2 | 0.5  ± 0.2 | 0.8  ± 0.1 | 0.5  ± 0.1 | 0.6  ± 0.1 | 0.7  ± 0.1 | 0.4  ± 0.1 | 0.7  ± 0.1 | 0.6  ± 0.1 |
| **Deoxyschisandrin** | 34.6  ± 3.1 | 43.2  ± 6.3 | 58.1  ± 1.3 | 45.1  ± 3.7 | 47.2  ± 3.7 | 56.4  ± 3.3 | 43.0  ± 3.4 | 53.4  ± 5.9 | 47.7  ± 5.7 |
| **γ-Schisandrin** | 4.7  ± 0.6 | 10.6  ± 2.0 | 15.5  ± 0.3 | 11.5  ± 0.2 | 12.2  ± 2.2 | 14.0  ± 2.1 | 10.4  ± 0.4 | 13.9  ± 0.2 | 12.3  ± 0.2 |
| **Schisandrin C** | 5.8  ± 3.2 | 2.8  ± 0.2 | 4.1  ± 0.1 | 2.8  ± 0.1 | 2.8  ± 0.1 | 3.6  ± 0.4 | 2.5  ± 0.1 | 3.9  ± 0.1 | 3.0  ± 0.2 |
| **Angeoyl-/tigloylgomisin H** | 36.4  ± 3.6 | 36.6  ± 3.3 | 46.8  ± 1.2 | 35.6  ± 0.9 | 35.0  ± 2.3 | 44.7  ± 4.1 | 33.1  ± 1.2 | 44.9  ± 5.9 | 35.9  ± 3.3 |
| **Angeoyl-/tigloylgomisin Q** | 76.8  ± 8.1 | 62.2  ± 6.0 | 8± 0.1  ± 6.5 | 6± 0.1  ± 4.2 | 57.6  ± 6.0 | 77.9  ± 5.2 | 56.9  ± 5.1 | 77.4  ± 5.5 | 65.4  ± 4.1 |
| **Schisandrin B** | 20.7  ± 3.3 | 24.5  ± 0.2 | 35.2  ± 1.2 | 25.9  ± 0.4 | 26.5  ± 3.6 | 30.2  ± 3.3 | 24.0  ± 0.3 | 32.1  ± 3.6 | 26.8  ± 3.7 |
| **Benzoylgomisin P** | 25.9  ± 3.8 | 22.6  ± 0.7 | 32.9  ± 0.9 | 23.2  ± 0.7 | 26.4  ± 0.9 | 27.8  ± 3.0 | 22.7  ± 0.8 | 29.8  ± 2.7 | 25.0  ± 1.0 |
| **Schisantherin D** | 8.9  ± 2.5 | 4.8  ± 0.4 | 8.1  ± 0.2 | 6.0  ± 0.3 | 6.1  ± 0.2 | 6.9  ± 0.6 | 5.6  ± 0.1 | 7.3  ± 0.2 | 5.8  ± 0.2 |
| **Total content** | **366.2**  **± 45.1** | **317.1**  **± 28.6*** | **427.8**  **± 25.1*** | **323.3**  **± 22.5*** | **324.8**  **± 31.1*** | **405.2**  **± 36.7*** | **301.9**  **± 21.6*** | **405.2**  **± 38.6*** | **336.9**  **± 31.1*** |

**Table S3.** Production [mg/100 g DW ± SD] of dibenzocyclooctadiene lignans in agitated microshoot cultures of *S. chinensis* elicitated on 23^rd^ and 27^th^ days of 30 days growth cycles with different concentrations of cadmium chloride – CdCl_2_ (mM). Control – agitated microshoots without CdCl_2_ elicitation. n ± 3, **p*<0.05 *vs* Control.

| **Lignans** | **Control** | **Elicitation scheme [day added /cadmium chloride – CdCl_2_ concentration (mM)]** | | | | | | | |
| --- | --- | --- | --- | --- | --- | --- | --- | --- | --- |
|  |  | **23/2.5** | **23/5** | **23/10** | **23/20** | **27/2.5** | **27/5** | **27/10** | **27/20** |
| **Schisandrin** | 80.5  ± 8.0 | 133.6  ± 8.4 | 107.1  ± 11.0 | 128.0  ± 11.3 | 143.0  ± 13.1 | 94.0  ± 8.2 | 103.8  ± 10.2 | 119.7  ± 8.3 | 100.9  ± 10.7 |
| **Gomisin A** | 52.8  ± 7.5 | 70.3  ± 5.2 | 54.4  ± 2.4 | 69.9  ± 3.4 | 77.9  ± 5.7 | 47.9  ± 1.4 | 51.9  ± 5.2 | 75.2  ± 4.1 | 52.7  ± 2.4 |
| **Gomisin G** | 3.9  ± 0.7 | 3.9  ± 0.4 | 5.1  ± 0.4 | 6.1  ± 0.4 | 8.0  ± 0.2 | 2.5  ± 0.9 | 2.9  ± 0.3 | 3.6  ± 0.4 | 3.8  ± 0.4 |
| **Schisantherin A** | 1.8  ± 0.2 | 3.0  ± 0.2 | 4.2  ± 1.2 | 5.1  ± 0.2 | 4.8  ± 0.3 | 2.4  ± 0.5 | 2.6  ± 0.3 | 2.7  ± 0.2 | 2.4  ± 0.5 |
| **Schisantherin B** | 10.8  ± 0.2 | 8.5  ± 0.7 | 8.3  ± 0.7 | 11.7  ± 0.5 | 11.3  ± 1.1 | 6.4  ± 0.3 | 7.4  ± 0.4 | 7.9  ± 0.5 | 6.6  ± 1.4 |
| **Schisanthenol** | 2.5  ± 0.2 | 1.3  ± 0.4 | 1.8  ± 0.9 | 6.3  ± 0.3 | 5.2  ± 0.4 | 1.1  ± 0.2 | 1.3  ± 0.1 | 1.5  ± 1.0 | 1.0  ± 0.2 |
| **Deoxyschisandrin** | 34.6  ± 3.1 | 79.9  ± 9.0 | 60.9  ± 5.2 | 69.9  ± 6.4 | 83.1  ± 6.2 | 61.4  ± 6.2 | 60.4  ± 5.0 | 77.0  ± 4.2 | 61.3  ± 3.2 |
| **γ-Schisandrin** | 4.7  ± 0.6 | 21.8  ± 2.7 | 17.0  ± 1.6 | 19.8  ± 2.4 | 22.7  ± 0.4 | 16.8  ± 4.0 | 18.0  ± 3.0 | 22.0  ± 2.4 | 16.8  ± 2.7 |
| **Schisandrin C** | 5.8  ± 3.2 | 6.2  ± 1.0 | 3.6  ± 0.4 | 4.5  ± 0.2 | 5.4  ± 0.2 | 4.2  ± 0.4 | 4.9  ± 0.7 | 5.6  ± 0.1 | 3.6  ± 1.0 |
| **Angeoyl-/tigloylgomisin H** | 36.4  ± 3.6 | 64.4  ± 3.2 | 55.8  ± 6.4 | 72.0  ± 4.7 | 74.7  ± 4.6 | 46.6  ± 4.0 | 54.3  ± 4.6 | 66.9  ± 4.1 | 49.5  ± 2.4 |
| **Angeoyl-/tigloylgomisin Q** | 76.8  ± 8.1 | 111.4  ± 10.4 | 85.3  ± 8.3 | 107.7  ± 9.7 | 120.0  ± 11.0 | 82.7  ± 9.0 | 93.3  ± 8.2 | 111.9  ± 14.0 | 80.5  ± 8.0 |
| **Schisandrin B** | 20.7  ± 3.3 | 43.0  ± 4.2 | 31.4  ± 3.2 | 39.4  ± 6.3 | 45.1  ± 2.4 | 33.1  ± 0.3 | 37.3  ± 6.0 | 41.9  ± 4.0 | 32.3  ± 3.0 |
| **Benzoylgomisin P** | 25.9  ± 3.8 | 41.1  ± 3.2 | 27.6  ± 2.3 | 36.9  ± 4.0 | 41.3  ± 4.2 | 29.1  ± 0.7 | 33.5  ± 2.0 | 39.7  ± 4.0 | 29.0  ± 1.3 |
| **Schisantherin D** | 8.9  ± 2.5 | 8.9  ± 1.0 | 6.0  ± 0.2 | 8.6  ± 0.4 | 10.1  ± 1.1 | 6.4  ± 0.1 | 7.3  ± 0.4 | 9.1  ± 1.0 | 6.4  ± 1.0 |
| **Total content** | **366.2**  **± 45.1** | **597.3**  **± 49.9*** | **468.5**  **± 44.1*** | **585.9**  **± 50.1*** | **652.5**  **± 50.8*** | **434.5**  **± 36.4*** | **478.9**  **± 46.4*** | **584.6**  **± 48.2*** | **446.8**  **± 38.0*** |

**Table S4.** Production [mg/100 g DW ± SD] of dibenzocyclooctadiene lignans in agitated microshoot cultures of *S. chinensis* elicitated on the first day of 30 days long growth cycles with different concentrations of cadmium chloride – CdCl_2_ (µM). Control – agitated microshoots without CdCl_2_ elicitation. n ± 3, **p*<0.05 *vs* Control.

| **Lignans** | **Control** | **Cadmium chloride – CdCl_2_ concentration (µM)** | | | | | | |
| --- | --- | --- | --- | --- | --- | --- | --- | --- |
|  |  | **1.25** | **12.5** | **125** | **250** | **500** | **1000** | **2500** |
| **Schisandrin** | 80.5  ± 8.0 | 109.4  ± 5.0 | 92.8  ± 21.8 | 89.1  ± 5.4 | 75.7  ± 16.0 | 87.4  ± 6.8 | 110.4  ± 10.2 | 128.2  ± 11.2 |
| **Gomisin A** | 52.8  ± 7.5 | 47.8  ± 1.8 | 31.5  ± 17.3 | 27.3  ± 2.4 | 16.8  ± 8.2 | 18.9  ± 4.5 | 34.0  ± 2.0 | 37.1  ± 8.2 |
| **Gomisin G** | 3.9  ± 0.7 | 8.8  ± 0.3 | 8.3  ± 2.7 | 6.7  ± 1.0 | 7.1  ± 1.6 | 6.2  ± 0.5 | 4.1  ± 0.4 | 4.2  ± 0.3 |
| **Schisantherin A** | 1.8  ± 0.2 | 3.5  ± 0.2 | 3.0  ± 0.8 | 3.7  ± 0.9 | 4.6  ± 1.5 | 4.8  ± 0.3 | 4.8  ± 0.5 | 3.6  ± 0.4 |
| **Schisantherin B** | 10.8  ± 0.2 | 12.1  ± 0.6 | 9.1  ± 2.4 | 12.1  ± 1.7 | 14.5  ± 3.7 | 19.3  ± 1.5 | 17.5  ± 1.0 | 12.1  ± 1.5 |
| **Schisanthenol** | 2.5  ± 0.2 | 4.4  ± 0.3 | 3.5  ± 0.4 | 3.2  ± 0.7 | 3.5  ± 1.0 | 3.8  ± 0.5 | 4.3  ± 0.3 | 2.8  ± 0.3 |
| **Deoxyschisandrin** | 34.6  ± 3.1 | 36.6  ± 2.5 | 29.4  ± 9.9 | 29.0  ± 4.0 | 27.0  ± 5.7 | 38.8  ± 5.4 | 55.1  ± 3.5 | 67.8  ± 8.1 |
| **γ-Schisandrin** | 4.7  ± 0.6 | 5.9  ± 0.3 | 4.7  ± 1.1 | 4.5  ± 0.6 | 4.3  ± 1.1 | 5.4  ± 0.3 | 7.4  ± 1.2 | 8.7  ± 2.4 |
| **Schisandrin C** | 5.8  ± 3.2 | 3.7  ± 1.2 | 2.2  ± 0.9 | 2.4  ± 0.2 | 1.9  ± 0.9 | 2.6  ± 0.7 | 4.2  ± 0.6 | 4.4  ± 0.5 |
| **Angeoyl-/tigloylgomisin H** | 36.4  ± 3.6 | 54.2  ± 1.8 | 48.3  ± 10.5 | 43.8  ± 4.1 | 39.8  ± 5.3 | 40.5  ± 5.3 | 49.7  ± 4.4 | 55.3  ± 6.8 |
| **Angeoyl-/tigloylgomisin Q** | 76.8  ± 8.1 | 95.5  ± 2.3 | 83.2  ± 17.2 | 80.5  ± 7.3 | 69.1  ± 11.9 | 76.3  ± 9.5 | 97.6  ± 16.7 | 107.5  ± 5.8 |
| **Schisandrin B** | 20.7  ± 3.3 | 24.1  ± 1.4 | 20.4  ± 3.8 | 19.4  ± 6.3 | 18.2  ± 1.6 | 21.8  ± 2.1 | 28.5  ± 4.9 | 33.5  ± 4.0 |
| **Benzoylgomisin P** | 25.9  ± 3.8 | 33.1  ± 1.9 | 29.7  ± 6.2 | 26.8  ± 4.2 | 23.8  ± 5.4 | 27.0  ± 2.7 | 36.8  ± 2.6 | 40.8  ± 6.8 |
| **Schisantherin D** | 8.9  ± 2.5 | 8.9  ± 1.0 | 8.4  ± 0.8 | 7.7  ± 0.9 | 7.1  ± 0.8 | 8.1  ± 0.9 | 9.0  ± 0.4 | 9.9  ± 0.5 |
| **Total content** | **366.2**  **± 45.1** | **448.1**  **± 20.7*** | **374.5**  **± 95.6*** | **356.3**  **± 39.4*** | **313.4**  **± 64.5*** | **360.7**  **± 40.9** | **463.4**  **± 48.6*** | **515.8**  **± 56.7*** |
